# The Eukaryotic Life on Microplastics in Brackish Ecosystems

**DOI:** 10.3389/fmicb.2019.00538

**Published:** 2019-03-20

**Authors:** Marie Therese Kettner, Sonja Oberbeckmann, Matthias Labrenz, Hans-Peter Grossart

**Affiliations:** ^1^Department of Experimental Limnology, Leibniz-Institute of Freshwater Ecology and Inland Fisheries, Berlin, Germany; ^2^Institute for Biochemistry and Biology, University of Potsdam, Potsdam, Germany; ^3^Environmental Microbiology Working Group, Leibniz Institute for Baltic Sea Research Warnemünde, Rostock, Germany

**Keywords:** microeukaryotes, plastic-associated biofilms, Baltic Sea, polyethylene, polystyrene, diversity profiles, network analysis, next-generation sequencing

## Abstract

Microplastics (MP) constitute a widespread contaminant all over the globe. Rivers and wastewater treatment plants (WWTP) transport annually several million tons of MP into freshwaters, estuaries and oceans, where they provide increasing artificial surfaces for microbial colonization. As knowledge on MP-attached communities is insufficient for brackish ecosystems, we conducted exposure experiments in the coastal Baltic Sea, an in-flowing river and a WWTP within the drainage basin. While reporting on prokaryotic and fungal communities from the same set-up previously, we focus here on the entire eukaryotic communities. Using high-throughput 18S rRNA gene sequencing, we analyzed the eukaryotes colonizing on two types of MP, polyethylene and polystyrene, and compared them to the ones in the surrounding water and on a natural surface (wood). More than 500 different taxa across almost all kingdoms of the eukaryotic tree of life were identified on MP, dominated by Alveolata, Metazoa, and Chloroplastida. The eukaryotic community composition on MP was significantly distinct from wood and the surrounding water, with overall lower diversity and the potentially harmful dinoflagellate *Pfiesteria* being enriched on MP. Co-occurrence networks, which include prokaryotic and eukaryotic taxa, hint at possibilities for dynamic microbial interactions on MP. This first report on total eukaryotic communities on MP in brackish environments highlights the complexity of MP-associated biofilms, potentially leading to altered microbial activities and hence changes in ecosystem functions.

## Introduction

Along with the exponential increase of plastic products in the past decades, the environmental pollution with plastic is constantly growing (Galloway et al., 2017; Geyer et al., 2017). Nowadays, “microplastics” (MP, plastic particles with a size below 5 mm) can be found in most aquatic environments (Eerkes-Medrano et al., 2015; Law, 2017), where they interact with organisms ranging from bacteria and algae to mammals (Zettler et al., 2013; Gall and Thompson, 2015; Clark et al., 2016). An important aspect of this pollution is that plastic provides an enormous surface for microbial colonization, and drifting MP can function as a vector for (micro)organism dispersal (Keswani et al., 2016). For a long time it is known that various eukaryotes such as diatoms and hydroids settle on MP (Carpenter and Smith, 1972). However, it took more than 30 years until the colonization of plastic received more scientific attention. Questions were raised whether MP could facilitate the spread of harmful algae (Masó et al., 2003), potential pathogens (Kirstein et al., 2016; Viršek et al., 2017), or invasive species (Barnes, 2002). The majority of studies in the field are focused on bacterial MP colonization (Hoellein et al., 2014, 2017; McCormick et al., 2014, 2016), whereas eukaryotic communities were often considered secondarily or analyzed solely microscopically, allowing for a relatively low coverage and taxonomic resolution (Masó et al., 2003; Carson et al., 2013; Zettler et al., 2013; Oberbeckmann et al., 2014; Reisser et al., 2014; Bryant et al., 2016). Though MP biofilms comprise a high number of different (micro) eukaryotes, solely a few systematic and detailed studies exist (Oberbeckmann et al., 2016; Debroas et al., 2017; Kettner et al., 2017). This scientific field is still at an early stage in describing the occurrence of (micro)eukaryotes on this anthropogenically introduced, artificial habitat and we are far away from understanding the ecological consequences, neither on local communities nor on the global ecosystem scale. Hence, a more holistic knowledge is required to better understand ecosystem and health related issues emerging from plastic pollution.

It has been shown that location, based on differences in environmental conditions, is one significant factor influencing the microbial community composition on MP (Hoellein et al., 2014; Oberbeckmann et al., 2014), emphasizing the need that studies should cover a wide range of ecosystems around the globe.

Our study area, the Baltic Sea, is one of the world’s largest brackish ecosystems. Its catchment area includes 14 countries with approximately 200 rivers and 85 million people. It is known, that rivers play a crucial role in transporting MP to seas and oceans (Lechner et al., 2014; Talvitie et al., 2015; McCormick et al., 2016; Lebreton et al., 2017; Mintenig et al., 2017). As the surface area of the Baltic Sea is four times smaller than its drainage area and since the average water residence time is three to four decades, its ecosystem suffers eminently from severe anthropogenic pressures such as eutrophication, chemical contamination, overfishing and intense shipping traffic (Snoeijs-Leijonmalm and Andrén, 2017). Today, the Baltic Sea ranks among the most polluted seas worldwide (HELCOM, 2010) and MP have emerged as additional anthropogenic pressure. Quantitative information on MP pollution in the Baltic Sea and its drainage basin, however, is scarce (Talvitie et al., 2015; Setälä et al., 2016; Lebreton et al., 2017). MP including synthetic fibers seem to be nearly omnipresent in samples from Baltic beaches in Germany (Stolte et al., 2015), Poland (Graca et al., 2017) and the Kaliningrad region (Esiukova, 2017). In the surface water of the River Warnow and Baltic Coast around Rostock, Germany, polyethylene (PE), and polystyrene (PS) are among the most commonly found types of MP (unpublished data, Leibniz Institute for Baltic Sea Research Warnemünde).

We did set up a 15-day incubation experiment, investigating the microbial colonization of two types of MP, namely PE and PS, in comparison with the natural surface wood in the Baltic Sea, the river Warnow, and a WWTP. Detailed analyses of the prokaryotic and fungal communities within these biofilms have already been published (Kettner et al., 2017; Oberbeckmann et al., 2018). Here, we now focus on the entire eukaryotic diversity. Beyond our project, prokaryotic and eukaryotic MP colonization in brackish ecosystems has not been investigated thoroughly.

We used results from Illumina MiSeq sequencing to test our hypothesis that eukaryotic MP-attached communities are distinct from communities on a natural surface (wood) and the surrounding water. Wood was chosen as a reference material since it occurs widely in natural aquatic systems, is degraded slowly and has a specific gravity similar to our plastics used. Further, we analyzed if the beta diversity is different on these tested substrate types and we evaluate whether eukaryotic assemblages differ among seven incubation sites located in a salinity gradient from the River Warnow into the Baltic Sea. We performed network analyses comprising prokaryotic and eukaryotic taxa to reveal which organisms co-occur and might interact with each other. Our detailed and holistic view into community compositions will provide new insights into the microbial life on MP in aquatic ecosystems and related consequences for ecosystem functioning.

## Materials and Methods

### Incubation Experiments

Polyethylene particles (PE, ExxonMobil^TM^ HDPE HTA 108, ExxonMobil Chemical Europe, Belgium, diameter 3–5 mm, density 0.96 g cm^-3^), polystyrene particles (PS, Polystyrol 143 E, BASF, Germany, diameter 3–5 mm, density 1.04 g cm^-^ł) and wood particles (1Heiz^®^Holzpellets, 1Heiz^®^Pellets AG, Germany, density 1.12 g cm^-3^) were exposed in triplicate to natural aquatic communities at seven different stations in north-east Germany. Particles were sampled after incubation for 15 days in surface water (1 to 3 m depth) in containers surrounded by a nylon mesh with 500 μm mesh size. Particles were rinsed with sterile station water and stored at -80°C until further processing. Additionally, we retrieved water samples on day 15 at each station for comparing the eukaryotic communities on the plastic substrates (PE, PS), the natural substrate wood, and the natural eukaryotic communities in the surrounding water. Water samples (1 to 3 replicates à 1-2 l) were filtered onto 3 μm pore-size membranes (Whatman^®^Nuclepore Track-Etch Membrane, polycarbonate, GE Healthcare, Germany) to concentrate the eukaryotes. The filtrate (2 to 3 replicates à 0.3-0.5 l) was subsequently filtered onto 0.22 μm pore-size membranes (Durapore^®^membrane filters, polyvinylidene fluoride, Merck Millipore Ltd., Ireland) to detect also the picoeukaryotes and eventual environmental DNA. Samples were stored at -80°C until further processing. We conducted the first incubation experiment in August/September 2014 at stations 1 to 5 (for map see Supplementary Figure S1). Station 1 is located at the pier Heiligendamm in the coastal Baltic Sea. Station 2 and 3 are situated close to the estuary mouth of the River Warnow, thereby station 2 is in the canal Alter Strom and station 3 in a marina on the other side of the estuary. Station 4 and 5 are located ca. 8 and 12 km, respectively, upstream in the River Warnow. The second incubation experiment was conducted in April/May 2015 in an anonymous wastewater treatment plant (WWTP). Station 6 is in the outlet of the last sedimentation treatment, where conventional WWTPs would discharge into the receiving waters. This WWTP has an additional treatment stage with an oxygenated biofilm reactor. Station 7 is located at the outlet of that reactor. Further details on the incubation experiments and sampling locations including environmental parameters and coordinates are given by Kettner et al. (2017).

### DNA Extraction, PCR Amplification, Sequencing and Sequence Processing

DNA extraction from PE, PS, wood, and filtered water samples was carried out based on a protocol published by Nercessian et al. (2005), which was optimized for our samples. The procedure includes a chemical, mechanical and enzymatic cell lysis step, followed by phenol-chloroform extraction and an ethanol precipitation of extracted nucleic acids. DNA was amplified by PCR using the universal eukaryote primers Eu565F and Eu981R (Stoeck et al., 2010; with addition of the bases -TGA at the 3′ end of the reverse primer according to LGC Genomics, Berlin, Germany), which target the highly variable V4 region of the 18S rRNA gene. Allowing for one mismatch, these primers cover 77.4% of al Eukaryota in the SILVA database v128 while excluding Bacteria and Archaea (Supplementary Table S1). PCR amplifications and subsequent sequencing on the Illumina MiSeq platform (2^∗^300 bp paired end, MiSeq reagent kit V3) were performed by LGC Genomics, Berlin, Germany. Raw Illumina reads were demultiplexed, then barcodes, adapters and primers were clipped. Reads were further processed in mothur v1.39.5 (Schloss et al., 2009; released in March 2017) following the mothur MiSeq SOP adapted to our target region (Kozich et al., 2013; url: https://www.mothur.org/wiki/MiSeq_SOP; online access May 2017). Processed reads were classified in mothur using SILVA’s non-redundant small subunit rRNA database v128 (Quast et al., 2013; released in September 2016). Taxonomy was based on the current SILVA taxonomy (Yilmaz et al., 2014; database v128) with the deepest possible taxonomic resolution at the genus level. Eukaryotic taxa are herein named according to their genus (for instance Ostreococcus) or – if no genus could be assigned – after the next higher classified level with the prefix “unclassified” (for instance unclassified Rhinosporidiosis). Further details on methods from DNA extraction to sequence processing were described previously by Kettner et al. (2017). Raw reads were made available under BioSample accessions from SAMN06806566 to SAMN06806660 of the BioProject PRJNA383789 at the Short Read Archive (SRA) of NCBI.

### Data Evaluation and Statistics

The final output is a read-abundance-table with all 22 taxonomic levels. The lists of the top 20 taxa per substrate were created based on relative abundances. After a transformation of the data (Legendre and Gallagher, 2001), bar charts were compiled on kingdom level for the different substrates types (PE, PS, wood, water >3.0 μm and 3.0 μm > water > 0.2 μm) and locations (station 1, 2, 3, 4, 5, 6, and 7). Second, we statistically evaluated the read-abundance-table for the deepest taxonomic levels using R (version 3.3.1, R Core Team, 2016) and the R package vegan 2.4-1 (Oksanen et al., 2016). To test whether the factors “substrate type” and “location” had a significant effect (*p* < 0.05) on the eukaryotic community composition, we performed a permutational multivariate analysis of variance (PERMANOVA) and pairwise PERMANOVA (adonis function in vegan, 999 permutations). Prior to that, the table was Hellinger-transformed (Legendre and Gallagher, 2001) and converted into a Bray-Curtis similarity matrix. A prerequisite for correct interpretation of PERMANOVA results is to check for homogeneity of dispersion, which we did with the betadisper and permutest function in vegan (999 permutations). A two-dimensional NMDS plot was created to visualize the Bray-Curtis similarity among the 95 samples. In addition, we calculated the Bray-Curtis similarity between each pair of substrates and stations, respectively. The eukaryotic diversity on different substrate types was calculated with the “ChaoJost” estimator for continuous diversity profiles (Chao and Jost, 2015) applying the Diversity function of the R package SpadeR version 0.1.1 (Chao et al., 2016). Before that, read abundances were added up for each substrate type and rarefied to 483071 reads to assure for comparability of diversity among substrates. Continuous diversity profiles (function ^q^Ḋ, see Chao and Jost (2015)) and specific Hill numbers (richness for *q* = 0, Shannon diversity for *q* = 1 and Simpson diversity for *q* = 2) were plotted with 95% confidence intervals using the R package ggplot2 version 2.2.1 (Wickham, 2009). To check if specific taxa were significantly associated with a single substrate type, we performed an “indicator species analysis” (R package indicspecies 1.7.6; De Cáceres and Legendre, 2009; De Cáceres and Jansen, 2016). Obtained *p*-values from multiple testing in pairwise PERMANOVA and indicator species analysis were adjusted according to Benjamini and Hochberg (1995).

### Phylogenetic Trees

Phylogenetic trees were compiled of the 20 most abundant taxa per substrate type. Representative sequences (most abundant sequence within the according taxon) were aligned using the SINA Aligner v1.2.11 (Pruesse et al., 2012) and phylogenetically analyzed using the ARB software package arb-6.0 (Ludwig et al., 2004) with the SILVA non-redundant small subunit rRNA database v132 (Quast et al., 2013), reduced to eukaryotic sequences. After adding the aligned sequences to the ARB database, the alignment was checked manually and the 305–390 bp long sequences along with their close relatives were used to calculate the trees. The phylogenetic relationships were deduced by the neighbor joining method, and bootstrap values were obtained by calculating 1000 bootstrap trees.

### Network Analysis

For the network analyses of PE-, PS-, and wood-associated biofilms, we combined both prokaryote (Oberbeckmann et al., 2018) and eukaryote datasets, which were independently Hellinger-transformed (Legendre and Gallagher, 2001) beforehand. Water samples were not included, as the objective was herein to characterize interaction possibilities within biofilm communities only. With regard to the strong differences in community composition, we calculated the networks separately for experiment I (River Warnow to Baltic Sea, stations 1–5, in total 15 samples per substrate type) and experiment II (WWTP, station 6 and 7, in total 6 samples per substrate type). Exclusively taxa, which occurred in at least half of the samples and had a relative abundance of more than 0.2% within the dataset, were used for the analysis. Network analyses were conducted in Cytoscape version 3.5.1 (Shannon et al., 2003) with the CoNet 1.1.1. beta application following the recommendations of Faust and Raes (2016). Taxa correlations were validated running networks with 1000 iterations. As we focused on possible co-occurrences, we chose only positive edges for network visualization. Topological parameters of co-occurrence networks were analyzed with the Network Analyzer tool release 2.7 (Assenov et al., 2008) in Cytoscape.

## Results

### Eukaryotic Communities Across Different Substrate Types and Locations

**FIGURE 1:**
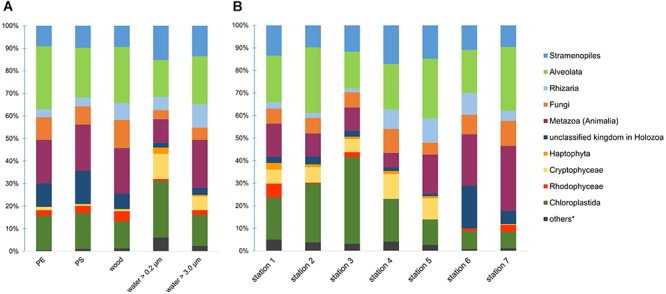
Eukaryotic community composition on kingdom level across different substrate types **(A)** and locations **(B)**. Proportions in bar charts are based on read counts after a Hellinger-transformation. ^∗^others = Amoebozoa, Discicristoidea, “Incertae Sedis” and an unclassified kingdom in Eukaryota.

From all 95 samples with more than 3.67 million reads, we were able to identify 738 different eukaryotic taxa. On PE and PS, we detected 426 and 433 different taxa, respectively. The 738 taxa were assigned to 14 different kingdoms. Common representatives of our samples were from the SAR supergroup (Stramenopiles + Alveolata + Rhizaria), Fungi, Holozoa including Metazoa, and different algae, especially Chloroplastida. The composition of kingdoms varied across the substrate types and locations (Figure 1). For instance, water samples had a higher proportion of Cryptophyceae, whereas PE and PS had more reads assigned to a kingdom within Holozoa, mainly from the order Rhinosporideacae. Compared to samples from the River Warnow and the Baltic Sea (stations 1 to 5), WWTP samples (stations 6 and 7) had almost no Cryptophyceae and Haptophyta, fewer Chloroplastida, but more Holozoa.

We compiled a list of the 20 most abundant taxa (by read counts) for a rough description of eukaryotic communities of the different substrate types (Supplementary Table S2). These top 20 taxa comprise ca. 70 to 81% of the respective community. Additionally, we calculated phylogenetic trees for all substrate types that show the closest relatives of these top 20 taxa, i.e., their representative sequence, respectively (Supplementary Figure S2). When comparing the top 20 taxa across the different substrate types (Supplementary Figure S2 and Table S2), we observe omnipresent taxa as well as taxa differentially abundant on one substrate or another. Among the top 20 taxa on PE and PS, we found organisms from different trophic levels. Green algae from the genus *Ulva* (sea lettuce) and the class Trebouxiophyceae were common primary producers in MP biofilms. As primary or secondary consumers, we detected different ciliates assigned to Peritrichia, the ConThreeP group, specifically *Zoothamnium* and the suctorian *Ephelota*. Consumers from the kingdom Metazoa (Animalia) were the rotifers Adinetida and Ploimida, the nematodes Diplogasterida and Rhabditida, the mollusk Caenogastropoda and the crustacean Podocopida. With the ability to retain kleptochloroplasts from their prey (Burkholder and Marshall, 2012), we found the dinoflagellate *Pfiesteria* as a potential temporary mixotroph. We further identified fungi from Chytridiomycota, for instance *Chytridium*, as well as fungal-like organisms such as Rhinosporideacae, *Rhizidiomyces*, and *Pythium*, which can have saprotrophic or parasitic life styles. Common organisms from the smaller water fraction (0.2 to 3.0 μm) were picoeukaryotic green algae such as *Ostreococcus* and *Micromonas*, the cryptophytes *Leucocitos* and *Teleaulax*, or the heterotrophic *Picomonas*, which were all rare on the solid substrates. In comparison to PE and PS, the larger water fraction (>3 μm) contained more phototrophs such as the diatoms *Skeletonema* and *Thalassiosira* or the green algae *Scenedesmus*. The compilation of the top 20 taxa on wood were similar to those on PE and PS, but included the fungus LKM11 among the dominant organisms.

**FIGURE 2:**
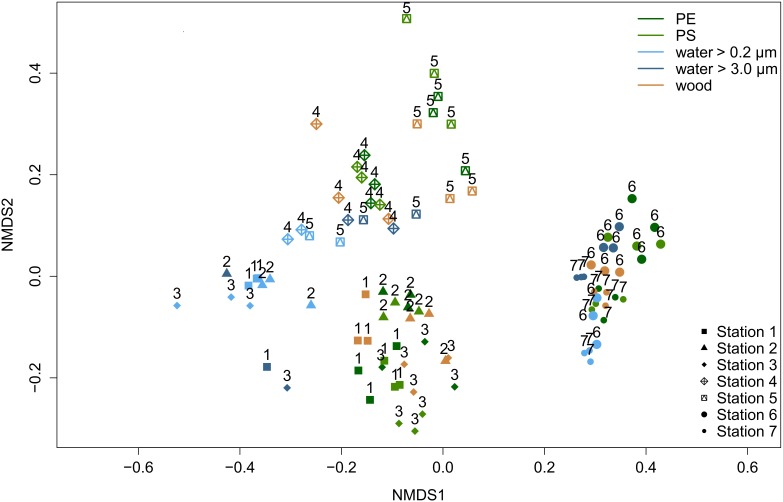
Non-metric multidimensional scaling (NMDS) ordination visualizing Bray-Curtis similarities of eukaryotic communities among individual samples (stress value = 0.15). With increasing similarity, the points have a closer proximity. Colors represent different substrate types. Symbols and numbers indicate different locations.

When comparing the eukaryotic communities at the deepest classified taxon level (ideally genus), we observed a significant impact of the factors substrate type (*p* = 0.001; *R*^2^ = 0.14), location (*p* = 0.001; *R*^2^ = 0.47) and their interaction (*p* = 0.001; *R*^2^ = 0.27) on the community composition. All results of the permutational multivariate analysis of variance, short PERMANOVA, are shown in Supplementary Table S3. A homogeneous data dispersion among the factor groups, which is necessary for a clear interpretation of PERMANOVA results, was given (Supplementary Table S4). We tested further with pairwise PERMANOVA (Supplementary Table S5), which substrates and locations differed from each other. The eukaryotic community differed significantly among all substrate types (*p* < 0.04; Supplementary Table S5), with the exception of PE and PS (*p* = 0.942; Supplementary Table S5). Communities on both MP types displayed a Bray–Curtis similarity of 78.7 % (Supplementary Table S5). The lowest Bray-Curtis similarities (41.8 to 46.2%; Supplementary Table S5) were observed between communities on the solid substrates PE, PS, and wood vs. the surrounding water communities (size fraction 0.2 to 3.0 μm). Also, each location had a significantly different community composition than any other station (*p* ≤ 0.005; Supplementary Table S5). We found the highest Bray-Curtis similarities between stations that were geographically close to each other, namely station 4 and 5 (70.4%; Supplementary Table S5), the two estuary stations 2 and 3 (65.5%; Supplementary Table S5) and both WWTP stations 6 and 7 (66.1%; Supplementary Table S5). The Bray–Curtis similarity matrix for individual samples is illustrated in a non-metric multidimensional scaling (NMDS) plot (Figure 2). The plot visualizes a grouping by location, a separation between the communities associated with solid substrates and water (lower similarity) and the proximity of MP-associated communities (higher similarity).

### Diversity of Eukaryotes on Different Substrate Types

**FIGURE 3:**
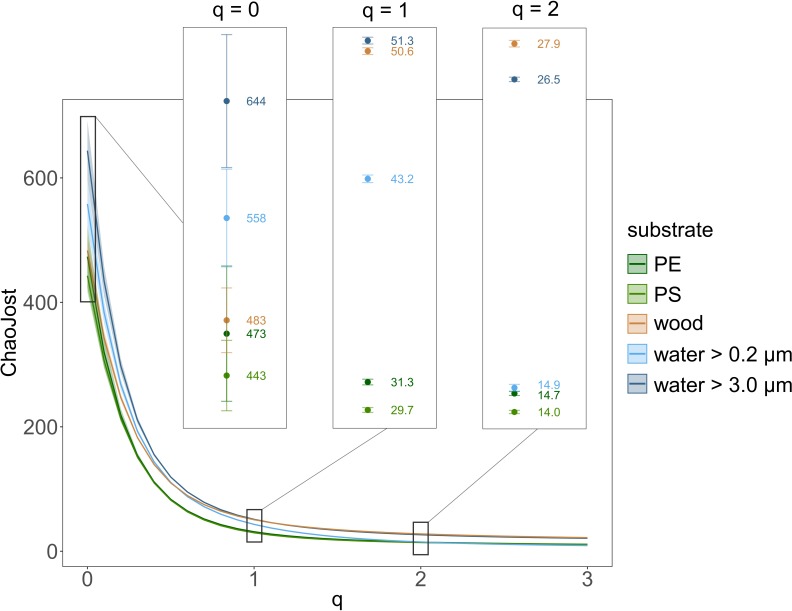
Eukaryotic taxon diversity presented as continuous diversity profiles with 95% confidence intervals for the substrates PE, PS, and wood as well as the small (between 0.2 and 3.0 μm) and large water size fraction (>3.0 μm). Diversity estimator “ChaoJost” (as proposed by Chao and Jost, 2015) on the *y*-axis and diversity order q on the *x*-axis. Estimated richness (*q* = 0), Shannon diversity (*q* = 1, exponential of Shannon entropy) and Simpson diversity (*q* = 2, inverse Simpson concentration) are enlarged in boxes.

Continuous diversity profiles for the different substrate types are depicted in Figure 3, with “ChaoJost” as the estimated diversity (^q^Ḋ) over the diversity order q (Chao and Jost, 2015). These profiles allow for a quick comparison of diversities, since, e.g., non-overlapping graphs indicate a higher diversity of the upper graph, i.e., the respective substrate type. The continuous diversity profiles comprise further three classical alpha-diversity estimators as special cases along the graph (as explained in legend of Figure 3). When comparing the estimated taxon richness (*q* = 0), both water size fractions were more diverse than the solid substrates PE, PS and wood. This ranking changes when we follow the profiles with increasing q, while the influence of rare taxa on diversity estimations decreases. For *q* ≥ 2, we still observe a lower taxon diversity of PE and PS, but the smaller size fraction in water (between 0.2 and 3.0 μm) had a similarly low diversity, whereas wood had an even higher diversity than the larger size fraction of water (>3.0 μm). Since diversity profiles and their 95% confidence intervals of the water size fraction >3.0 μm and those of PE and PS never overlapped, we can conclude that eukaryotic communities in water (>3 μm) had a significantly higher diversity than on MP.

### Potential Eukaryotic Key Taxa

We conducted an “indicator species analysis” (De Cáceres and Legendre, 2009) to identify eukaryotic taxa, which were significantly more abundant on certain substrate types. Only one taxon each was associated with PE and PS, a rotifer and a chlorophyte (Supplementary Table S6). Wood-associated taxa were assigned mainly to Fungi from the phylum Ascomycota or to Alveolata from the phylum Ciliophora (Supplementary Table S6). The lists of taxa that were associated with water were substantially longer and comprised a greater variety of eukaryotic kingdoms. Several small eukaryotes were associated with the smaller water fraction (between 0.2 and 3.0 μm), such as *Ochromonas* (Ochrophyta), *Picomonas* (Picozoa), *Micromonas*, and *Ostreococcus* (both Chlorophyta), or *Geminigera* and *Teleaulax* (both Cryptomonadales) (Supplementary Table S6). Associated with the larger water fraction (>3.0 μm) were a number of nematodes and arthropods, many different chlorophytes, for instance *Monoraphidium* and *Scenedesmus*, or ochrophytes such as *Thalassiosira*, *Cyclotella*, and *Nannochloropsis*, as well as many other taxa across different phyla (Supplementary Table S6).

*Pfiesteria* was the most abundant genus (by reads counts) on PE and the second most abundant on PS. It was detected mainly at the stations 4 and 5 (together 99.7%; Supplementary Tables S7, S8). Read counts from *Pfiesteria* originated with more than 88% from MP (PE+PS) and less than 2% from water (Supplementary Table S7). This signifies a strong enrichment of *Pfiesteria* on MP and indicates a preference toward these substrate types. To obtain more information about potential relatives of *Pfiesteria*, a representative sequence (most abundant read; get.oturep function in mothur) was checked with the NCBI’s blastn program (BLASTN 2.6.1, default settings; Zhang et al., 2000; Morgulis et al., 2008). The top 50 search results are presented in Supplementary Table S9. Among those, 10 hits were *Pfiesteria piscicida*, with 100% query coverage and 99% identity to our representative sequence. The close phylogenetic relation is further supported by the positioning of our representative sequence next to *Pfiesteria piscicida* in our calculated phylogenetic trees (Supplementary Figure S2).

### Co-occurrence Networks

**FIGURE 4:**
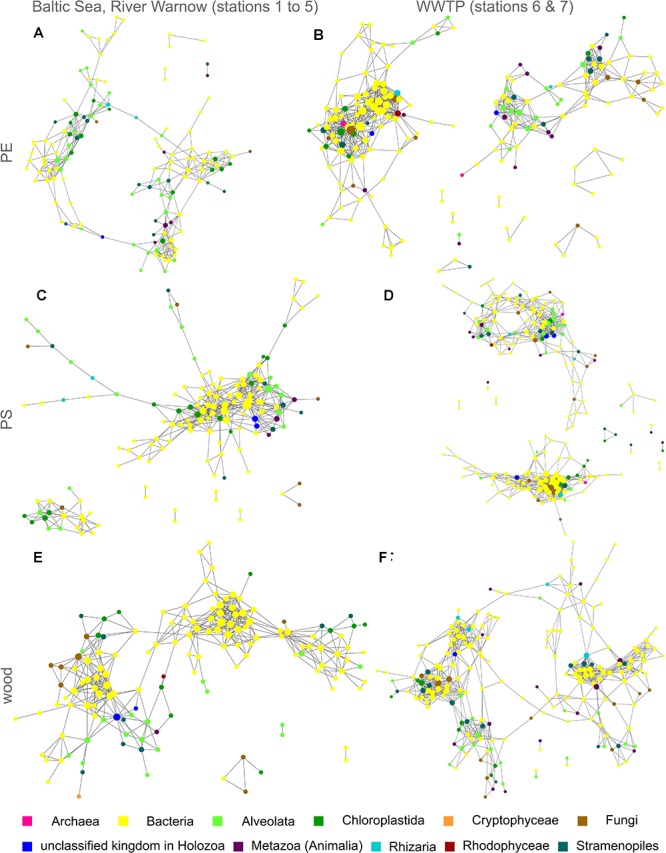
Co-occurrence networks of prokaryotic and eukaryotic taxa for PE, PS, and wood from both incubation experiments I (Baltic Sea to River Warnow, stations 1 to 5) and II (WWTP, stations 6 and 7). Each node is a taxon and the node diameter increases with the number of direct neighbors. Edges represent significant positive correlations (*p* < 0.05) between nodes/taxa. Colors indicate different kingdoms. **(A)** PE in Baltic Sea, River Warnow (stations 1 to 5); **(B)** PE in WWTP (stations 6 and 7); **(C)** PS in Baltic Sea, River Warnow (stations 1 to 5); **(D)** PS in WWTP (stations 6 and 7); **(E)** Wood in Baltic Sea, River Warnow (stations 1 to 5); **(F)** Wood in WWTP (stations 6 and 7).

To evaluate interaction possibilities among taxa within communities on the different solid substrate types PE, PS, and wood, we constructed co-occurrence networks, which contain not only eukaryotic but also prokaryotic taxa (Figure 4 and Supplementary Figure S3 with nodes labeled with taxon names). Each node of a network represents a different taxon and the edges are significant positive correlations between the nodes/taxa. For all substrate types, we observed numerous positive correlations among prokaryotes, eukaryotes as well as between prokaryotic and eukaryotic taxa, particularly in the WWTP (Figure 4, Table 1, and Supplementary Figure S3). Beside the variety of bacterial taxa, eukaryotic taxa of the kingdoms Chloroplastida, Alveolata and Stramenopiles dominated within the co-occurrence networks. Especially on PE and PS, bacteria appeared to be highly interconnected with eukaryotes (Figure 4A–D), whereas on wood bacteria were primarily correlated to other bacteria (Figure 4E). Fungi were represented more often on wood and all substrate types inside the WWTP (Figure 4B,D,F), than in networks of PE or PS in the Baltic Sea and River Warnow (Figure 4A,C). Archaea occurred very rarely and exclusively in WWTP networks (Figure 4B,D). All networks are highly heterogeneous and on average rather decentralized, meaning that only few nodes have a central position within the network (Table 1). This is also reflected in the formation of several clusters (denser grouping of nodes) within networks, resulting often in entirely dis-connected clusters (see Table 1 for number of connected components, wherein a connected component is defined as a cluster in which all nodes are directly or indirectly, i.e., via further nodes, connected to each other). For instance, in the WWTP, both PE and PS networks formed two large dis-connected clusters and additionally some smaller clusters (Figure 4B,D). When looking at the respective taxa within these large clusters, it became apparent that those taxa in the left cluster for PE (Figure 4B and Supplementary Figure S3B) and in the lower cluster for PS (Figure 4D and Supplementary Figure S3D) were more abundant in station 6, whereas the other taxa dominated in stations 7. The same location-dependent formation of clusters holds true for the other networks.

**Table 1:** Topological parameters of co-occurrence networks for PE, PS, and wood from both incubation experiments I (Baltic Sea to River Warnow, stations 1 to 5) and II (WWTP, stations 6 and 7).

	PE stations 1–5	PE stations 6–7	PS stations 1–5	PS stations 6–7	Wood stations 1–5	Wood station 6–7
input taxa	188	225	188	239	195	228
number of nodes (output taxa taxa)	134	196	144	207	144	208
number of edges	314	700	416	823	451	776
clustering coefficient	0.38	0.48	0.38	0.41	0.45	0.46
connected components	4	10	7	11	4	4
network diameter	15	10	13	12	13	16
network centralization	0.06	0.12	0.17	0.10	0.10	0.06
network heterogeneity	0.67	0.85	0.88	0.85	0.74	0.69
shortest paths	16012	15000	13050	17148	18376	40608
shortest paths (in percent)	89%	39%	63%	40%	89%	94%
average shortest path length	5.98	3.63	4.13	3.58	5.02	6.19
average number of neighbors	4.69	7.14	5.78	7.95	6.26	7.46


*Networks were analyzed in cytoscape version 3.5.1 (Shannon et al., 2003) with the Network Analyzer tool release 2.7 (Assenov et al., 2008)*.

## Discussion

### Effect of Substrate Type and Location on Eukaryotic Communities

Our results support the hypothesis that substrate type has a significant impact on eukaryotic community composition in aquatic systems. Eukaryotic communities on MP differed from those on floating wood and in the surrounding water. The lowest similarities were found between communities of the smaller water size fraction (0.2 to 3.0 μm) and solid substrates (PE, PS, and wood) and might be a result of the lifestyle of certain picoeukaryotes dominating in these water samples. Small organisms such as *Ostreococcus* and *Micromonas* have a high surface to volume ratio, which is advantageous for the uptake of nutrients, i.e., they are well adapted for living freely in the water column and this could explain their lower abundances on the solid substrates. Distinct differences between communities on MP, on natural surfaces, and in water have been shown previously also for bacterial and fungal communities from the same experimental set-up (Kettner et al., 2017; Oberbeckmann et al., 2018) as well as in other marine and freshwater studies (Hoellein et al., 2014; McCormick et al., 2014, 2016; Oberbeckmann et al., 2016). This suggests that plastic affects both prokaryotic and eukaryotic community compositions alike. No significant differences were detected between the eukaryotic communities on PE and PS, which is in accordance with other studies comparing microbial communities between plastic and other hard substrates (Hoellein et al., 2014; Oberbeckmann et al., 2016). Despite low sample replication, other studies, however, hint at distinct microbial colonization patterns among different plastic/polymer types (Zettler et al., 2013; Debroas et al., 2017). Plastic-associated communities also differ from organic substrates (Hoellein et al., 2014; McCormick et al., 2014, 2016), which is confirmed by our comparison of communities on MP vs. wood.

Besides the outlined effect of substrate type, we identified a strong impact of location on eukaryotic community composition. Location-dependency of plastic-associated microbial communities has been observed previously (Hoellein et al., 2014; Oberbeckmann et al., 2014, 2016, 2018; Amaral-Zettler et al., 2015; Kettner et al., 2017) and as it is generally accepted that local environmental factors are influencing microbial colonization patterns, we will not further discuss this in more detail.

In general, the existing studies on microbial plastic colonization are difficult to compare, especially due to differences in sampling environments, seasons, plastic types, biofilm age or approaches to identify species. Nevertheless, we can see several similarities, i.e., the occurrence of diatoms as early colonizers, a high frequency of organisms from the SAR-group including dinoflagellates and the suctorian *Ephelota*, as well as different algae and holozoans (Carpenter and Smith, 1972; Masó et al., 2003; Carson et al., 2013; Zettler et al., 2013; Hoellein et al., 2014; Bryant et al., 2016; Oberbeckmann et al., 2016; Debroas et al., 2017). Despite the small particle sizes of MP, we identified numerous organisms assigned to the kingdom Metazoa/Animalia such as nematodes, rotifers or annelids suggesting they attach to MP mainly as eggs, larvae and juveniles, or their environmental DNA (eDNA) adsorbed to the particle. We assume that the retrieved high read abundances of metazoans might rather reflect their multicellularity than the actual number of individuals. In contrast to previous studies, our incubation experiment revealed a remarkably diversity fungal taxa (Kettner et al., 2017), and enabled us to report here for the first time a *Pfiesteria*-related dinoflagellate as the dominant taxon on MP. The high sequencing depth and sample number allowed us to capture a higher eukaryotic diversity and at the same time, we obtained a deeper taxonomic resolution than previous studies.

### Diversity and Co-occurrence Patterns of Microorganisms on MP

Taking together all MP samples, we identified more than 500 different eukaryotic taxa and the majority of eukaryotic kingdoms from the tree of life were present. Yet, the eukaryotic diversity was significantly lower on MP than on wood or in the surrounding water (>3.0 μm). Also in other studies, MP communities were found to be less diverse than in water (Zettler et al., 2013; Debroas et al., 2017). As we detected, respectively, solely one taxon that was specifically associated with PE or PS and due to the lower diversity, we assume that MP was colonized mainly by opportunistic eukaryotes. Possibly, PE and PS rather excluded organisms than attracting a specialized MP community. Maybe a longer incubation time would lead to more mature biofilms with possibly more micro-niches for a number of additional organisms. Though wood was exposed over the same time span, it showed the highest eukaryotic diversity. The higher attractiveness of wood for microeukaryotic colonization compared to MP may have been caused by its rougher surface facilitating microbial cell attachment. Additionally, bacteria and fungi can utilize wood as a substrate source, which renders nutrients available also to other organisms and thus increases organismic diversity. Other researchers provide hints, that also plastic might be degraded to a certain degree by the attached bacterial and fungal community (Zettler et al., 2013; McCormick et al., 2016; Debroas et al., 2017). Although we did not check for MP bio-degradation, we assume it is an inferior process, since easier accessible nutrient and carbon sources are available in the MP biofilms - mainly provided by photoautotrophic algae.

Biofilms on MP and wood harbored various organisms simultaneously, which suggests a number of possible interactions such as symbiosis, predator-prey relationships, infections or the collective degradation of organic matter. Indeed, our network analyses revealed many positive correlations among eukaryotes and especially among bacteria, as well as between bacteria and different eukaryotic kingdoms. For instance, the numerous positive correlations between fungal and bacterial taxa on wood could support the idea of a collective metabolization of this substrate. *Amoebophyra*, a dinoflagellate known to infect other dinoflagellates (Kim et al., 2008), was positively correlated to the occurrence of Suessiaceae on PE and another unclassified dinoflagellate on PS, which could indicate a parasitic relationship. The positive correlations on PE and PS of the suctorian *Ephelota* and different bacterial taxa might be explained by ectosymbiosis, which has been previously observed microscopically on MP by Zettler et al. (2013), interestingly, even with a sulfite-oxidizing bacterium. On PS, the picoeukaroyte *Micromonas* was positively correlated to the bacterium *Eudoraea* (family Flavobacteriaceae) and on PE to another bacterium of the order Flavobacteriales as well as to *Litoreibacter* (familiy Rhodobacteraceae). In experimental studies, several Flavobacteria and Alphaproteobacteria (including Rhodobacteraceae) were able to assimilate *Micromonas*-derived proteins (Orsi et al., 2016), which could hint at interactions between these organisms for the cycling of nitrogen also in our experiment. Beside bacteria, *Micromonas* was further correlated to protists. While this picoeukaryote is mainly grazed by dinoflagellates in open seawater (Orsi et al., 2018), it might have been consumed by ciliates in biofilms since *Micromonas* co-occurred with *Vorticella*, *Zoothamnium*, and *Holosticha* on PE. A significant co-occurrence can unfortunately only indicate but not prove for a microbial interaction (Faust and Raes, 2012). Certainly, many organisms simply co-exist together as they prefer similar environmental niches, e.g., the photosynthetic bacterium *Erythrobacter*, which occurred on PE and PS together with the algae *Picochlorum*. Although PE- and PS- associated community compositions had a high Bray-Curtis similarity, it seems challenging to identify many similarities in their co-occurrence patterns. This might give a first hint that other interaction possibilities exist on PE and PS, even though their biofilm communities are overlapping. Moreover, on PE and PS bacterial communities were more tightly linked to eukaryotic communities than on the natural substrate wood. This interesting finding hints to the fact that both plastic types do not serve as a bacterial substrate source rather than surfaces for colonization. The tight linkage of prokaryotic with eukaryotic communities implies that bacterial communities on MP are to a large extent controlled by the dynamics of (micro)eukaryotes, both as a substrate source and interaction partner. Another relevant observation from the PE, PS and wood co-occurrence networks was the obvious cluster-formation by sampling locations. This highlights that not merely the substrate type, but rather the locations – i.e., the local environmental factors – influenced the community compositions as well as their associated patterns of taxon co-occurrence, meaning their interaction possibilities.

### Relevance of MP Colonization for Aquatic Ecosystems

A plastic item, with a mass of one gram, floating in the open sea can harbor significantly more organismic biomass compared to one thousand liter of surrounding seawater (Mincer et al., 2016). Hence, the colonization of floating MP can have various implications for aquatic ecosystems.

First, we observed a strong enrichment of *Pfiesteria* reads on MP in comparison to the surrounding water. The respective sequence was closely related to the species *Pfiesteria piscicida*, which is able to produce toxins (Moeller et al., 2007) and is associated with harmful blooms and major fish kills (Glasgow et al., 2001). Toxic *Pfiesteria* strains can harm fishes by the toxin release itself, but also by physically attacking the epidermis (Burkholder and Marshall, 2012). Further, *Pfiesteria piscicida* is able to form resting stages (Coyne et al., 2006), which is particularly relevant for the survival and transport on MP to habitats far away. Also the dinoflagellate *Heterocapsa* was present on MP. Certain species within the *Heterocapsa* genus are able to form toxic red tides, which can cause mass mortality of bivalves (Horiguchi, 1995). Moreover, we detected numerous potentially parasitic eukaryotes such as zoosporic fungi (Kettner et al., 2017) or the Peronosporomycetes (former Oomycetes) *Pythium* and *Lagenidium*. Unfortunately, the short fragment length required for Illumina amplicon sequencing does not provide a resolution to species or strain level and is not giving any information on the life stage, making predictions of toxicity or infection potentials difficult. Nevertheless, we show that various potentially harmful eukaryotes or their close relatives can colonize and even enrich on MP. Consequently, our results support findings from Masó et al. (2003), who suggested plastic debris as a vector for harmful algal bloom species in marine environments.

Second, we need to consider the persistence of MP due to extremely low degradation rates (Shah et al., 2008; Andrady, 2011) compared to naturally occurring particles such as fecal pellets, algal aggregates or driftwood. MP offers a durable dispersal medium for the above mentioned harmful organisms as well as potential human pathogens (Kirstein et al., 2016), fish pathogens (Viršek et al., 2017), or bloom-forming dinoflagellates (Masó et al., 2003), potentially transporting them with ocean currents over thousands of kilometers (Law et al., 2014). This poses a serious threat to aquaculture (Horiguchi, 1995; Moestrup et al., 2014), wildlife, and humans. Barnes (2002) estimated that human litter, whereof the majority is plastic, more than doubles the rafting opportunities for all kinds of organisms, which may threaten the global marine biodiversity. For the Baltic Sea, maritime transport is currently the most important factor for the introduction of non-indigenous species (Oesterwind et al., 2016). Our study highlights that also MP has to be considered as an additional and even more frequent transport medium for numerous species in the Baltic Sea.

Third, floating colonized MP has the potential to change carbon, nutrient and energy dynamics in the aquatic realm. Recently, Yokota et al. (2017) reported on increased photosynthetic activities of cyanobacteria on MP. Since we detected numerous different algae on MP, we can also assume an increase in eukaryotic photosynthetic activity following MP colonization. Bryant et al. (2016) concluded that MP creates net autotrophic hot spots in the oligotrophic sea. Furthermore, the colonization of MP (Kaiser et al., 2017) as well as the incorporation of MP into algal aggregates (Long et al., 2015) or zooplankton fecal pellets (Cole et al., 2016) alters the overall load, leading either to enhanced sinking or floating of particles. Since organic aggregates represent the main vehicles for transport of organic matter from the sea surface to the bottom (Ducklow et al., 2001), MP has the potential to affect the oceanic carbon pump and vertical fluxes of nutrients.

## Conclusion

Our study demonstrates that MP biofilms in brackish and freshwater ecosystems comprise complex communities representing several trophic levels and interaction possibilities between them. When judging the potential ecological impact and the risk of dispersal of invasive or harmful organisms, the complexity and dynamic nature of MP biofilms have to be considered, especially in terms of their location-dependency. Our study provides evidence that biodiversity on MP is limited compared to natural surfaces, and that potential pathogens and parasites can thrive and even enrich on plastic surfaces. In the future, studies on survival rates during MP migration as well as systematic risk assessments regarding the impact of MP on biodiversity and infection potentials as well as ecosystem functions are vitally needed.

## Originality-Significance Statement

The pollution of aquatic environments with microplastics is of increasing relevance due to the high persistence toward mineralization, leading to global debris accumulation with potential effects on organisms of all trophic levels. Microplastics can serve as transport vehicles for attached microbes including potentially invasive or harmful organisms, with yet unknown consequences for wildlife. So far, the colonization of microplastics in brackish waters has been understudied and little attention has been paid to microeukaryotic colonizers. Herein, we present a detailed report on eukaryotic community compositions in the Baltic Sea, a river and a wastewater treatment plant. Based on next-generation sequencing results, we show how microplastic-associated eukaryotic communities differ from natural communities in water or on wood regarding their composition and diversity. Moreover, this is the first study providing co-occurrence networks for polyethylene, polystyrene and wood, while including prokaryotes as well as eukaryotes, highlighting their vast interaction possibilities. Taking all results together, we provide an in-depth view into microplastic-associated assemblages while discussing their potential environmental implications, which will push forward our understanding of the eukaryotic life in microplastic biofilms.

## Data Availability

Raw reads were made available under BioSample accessions from SAMN06806566 to SAMN06806660 of the BioProject PRJNA383789 at the Short Read Archive (SRA) of NCBI; https://www.ncbi.nlm.nih.gov/sra/?term=PRJNA383789. All datasets, which are not included in this article or Supplementary Material, will be made available by the authors upon request, without undue reservation, to any qualified researcher.

## Author Contributions

SO, ML, and H-PG created the project idea. The study was designed by SO and ML. Sampling was performed by SO and MTK. For eukaryotes, MTK was processing the samples and evaluated the datasets including bioinformatics and statistics. For prokaryotes, SO was processing the samples including bioinformatics. Networks for eukaryotes and prokaryotes were calculated by MTK. MTK prepared figures, tables, and Supplementary Material. The manuscript was written by MTK and revised by SO, ML, and H-PG.

## Conflict of Interest Statement

The authors declare that the research was conducted in the absence of any commercial or financial relationships that could be construed as a potential conflict of interest.
