# Supplementary material for: The Eukaryotic Life on Microplastics in Brackish Ecosystems
Source: Front Microbiol. 2019 Mar 20;10:538. doi: 10.3389/fmicb.2019.00538 (PMC6435590; doi:10.3389/fmicb.2019.00538)
Supplement: Supplementary file 1 [file Data_Sheet_1.PDF]

**Supplementary Material**

# The eukaryotic life on microplastics in brackish ecosystems

Marie Therese Kettner [1,2], Sonja Oberbeckmann [3], Matthias Labrenz [3],
Hans-Peter Grossart [*,1,2]

1 Department of Experimental Limnology, Leibniz-Institute of Freshwater Ecology and Inland Fisheries, Berlin, Germany

2 Institute for Biochemistry and Biology, Potsdam University, Potsdam, Germany

3 Environmental Microbiology Working Group, Leibniz Institute for Baltic Sea Research Warnemünde, Rostock, Germany

## Tables

**Table S1.** Primer specificity tested with Silva TestPrime1.0 (Klindworth et al., 2013). Forward primer: Eu565F CCAGCASCYGCGGTAATTCC, reverse primer: Eu981R ACTTTCGTTCTTGATYRATGA (Stoeck *et al.*, 2010; with addition of the bases -TGA by LGC Genomics, Berlin, Germany). List of taxa and their according coverage in the Silva Taxonomy browser (database v128). Coverage is given in percent allowing zero and one mismatch. ..... 4

**Table S2.** Lists of top 20 eukaryotic taxa on polyethylene (PE), polystyrene (PS), wood, and both water size fractions (water > 0.2 µm and water > 3.0 µm). Taxa are ranked by their read counts. Proportion of taxon reads to total reads (of the according substrate) are given in percent. ..... 5

**Table S3.** Results of permutational multivariate analysis of variance (PERMANOVA) after 999 permutations for the factors substrate and location as well as their interaction term. df = degrees of freedom, Sq = squares ..... 12

**Table S4.** Results of permutation tests for homogeneity of multivariate dispersions for different substrate types and locations. P-values over 0.05 indicate a homogenous dispersion. df = degrees of freedom, Sq = squares ..... 12

**Table S5.** Results of pairwise PERMANOVA (999 permutations) and Bray-Curtis (BC) similarity for different substrate types and locations. P-adjustment according to Benjamini and Hochberg, 1995. .......... 13
**Table S6.** Results of indicator species analysis with taxa associated to each substrate type. For each taxon the corresponding phylum, kingdom, indicator value and adjusted p-value (according to Benjamini and Hochberg, 1995) are given. Taxonomic information is based on SILVA (Yilmaz et al., 2014) database v128. .......... 14
**Table S7.** *Pfiesteria* read counts and percentages from different substrates and locations. . 20
**Table S8.** *Pfiesteria* read counts from the substrate replicates at station 4 and 5. .......... 20
**Table S9.** Top 50 hits from NCBI's Web BLAST service (BLASTN 2.6.1, default settings, (Morgulis et al., 2008; Zhang et al., 2000)) of the most abundant sequence that was assigned to the genus *Pfiesteria*. .......... 21

<u>Figures</u>

**Fig. S1.** On this map, study locations from the first incubation experiment (stations 1 to 5) are displayed. Station 6 and 7 from the second incubation experiment inside the wastewater treatment plant are not shown to preserve anonymity. The map was created using the R package leaflet (Cheng et al., 2017) and maps from © OpenStreetMap and d-maps.com, URL http://d-maps.com/m/europa/germany/allemagne/allemagne07.gif Map is published in black and white in Kettner et al., 2017. .......... 3
**Fig. S2.** Phylogenetic trees of representative sequences of the 20 most abundant taxa (in bold and with rank/position in top 20 list, see also Table S2) and their close relatives for each substrate type. Scale bar represents 10 nucleotide substitutions per 100 nucleotides. GenBank accession numbers and bootstrap values are displayed. .......... 7
**Fig. S3.** Co-occurrence networks for PE (A, B), PS (C, D) and wood (E, F) for both incubation experiments I (Baltic Sea to River Warnow, stations 1 to 5) and II (WWTP, stations 6 and 7). Networks were calculated in Cytoscape 3.5.1 (Shannon et al., 2003) with the CoNet app version 1.1.1. beta (Faust and Raes, 2016). Nodes are labelled with taxon name and relative node sizes correspond to number of neighbor nodes. Colors indicate different kingdoms. .. 24

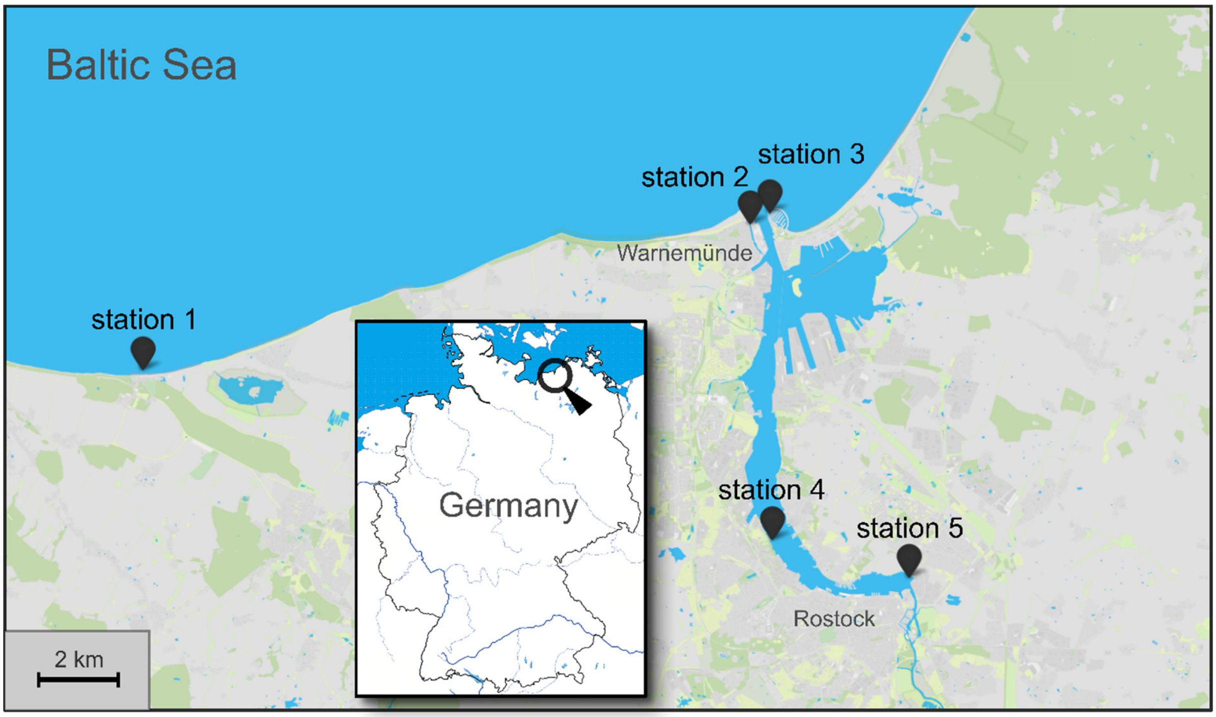


**Fig. S1.** On this map, study locations from the first incubation experiment (stations 1 to 5) are displayed. Station 6 and 7 from the second incubation experiment inside the wastewater treatment plant are not shown to preserve anonymity. The map was created using the R package leaflet (Cheng et al., 2017) and maps from © OpenStreetMap and d-maps.com, URL http://d-maps.com/m/europa/germany/allemagne/allemagne07.gif

Map is published in black and white in Kettner et al., 2017.

**Table S1.** Primer specificity tested with Silva TestPrime1.0 (Klindworth et al., 2013). Forward primer: Eu565F CCAGCASCYGCGGTAATTCC, reverse primer: Eu981R ACTTTCGTTCTTGATYRATGA (Stoeck *et al.*, 2010; with addition of the bases -TGA by LGC Genomics, Berlin, Germany). List of taxa and their according coverage in the Silva Taxonomy browser (database v128). Coverage is given in percent allowing zero and one mismatch.

| Taxon | coverage allowing zero mismatch | coverage allowing one mismatch | in our data-set |
|---|---|---|---|
| Eukaryota | 55.9% | 77.4% | yes |
| Chloroplastida | 79.9% | 86.9% | yes |
| Rhodophyceae | 88.6% | 100.0% | yes |
| Glaucophyta | 50.0% | 94.3% | no |
| Cryptophyceae | 85.7% | 96.0% | yes |
| Amoebozoa | 56.3% | 77.5% | yes |
| Haptophyta | 0.9% | 94.6% | yes |
| Incertae Sedis kingdom | | | |
| phylum Ancyromonadida | 100.0% | 100.0% | yes |
| unclassified kingdom in Holozoa | | | |
| phylum Choanoflagellida | 86.0% | 95.2% | yes |
| phylum Ichthyosporea | 21.7% | 88.3% | yes |
| Metazoa (Animalia) | 62.1% | 81.3% | yes |
| Excavata | 2.8% | 13.2% | no |
| Centrohelida | 94.7% | 95.8% | no |
| Discicristoidea | 60.4% | 89.6% | yes |
| Fungi | 19.2% | 59.1% | yes |
| Stramenopiles | 87.8% | 93.7% | yes |
| Alveolata | 73.4% | 88.0% | yes |
| Rhizaria | 0.2% | 53.5% | yes |
| unclassified kingdom in Eukaryota | | | |
| phylum Picozoa | 89.5% | 92.1% | yes |
| phylum DH147-EKD10 | 72.7% | 90.9% | yes |
| phylum SA1-3C06 | 0.0% | 100.0% | yes |
| Bacteria | 0.0% | 0.0% | no |
| Archaea | 0.0% | 0.0% | no |

**Table S2.** Lists of top 20 eukaryotic taxa on polyethylene (PE), polystyrene (PS), wood, and both water size fractions (water > 0.2 µm and water > 3.0 µm). Taxa are ranked by their read counts. Proportion of taxon reads to total reads (of the according substrate) are given in percent.

| **PE** | **reads** | **percent** | **PS** | **reads** | **percent** | **wood** | **reads** | **percent** |
|---|---|---|---|---|---|---|---|---|
| Pfiesteria | 117566 | 14.5% | unclassified Rhinosporideacae | 114099 | 13.8% | Ephelota | 45773 | 9.5% |
| unclassified Peritrichia II | 116312 | 14.3% | Ulva | 106131 | 12.8% | unclassified ConThreeP | 31940 | 6.6% |
| Ulva | 85737 | 10.6% | Pfiesteria | 98750 | 11.9% | unclassified Peritrichia II | 26399 | 5.5% |
| unclassified Adinetida | 55483 | 6.8% | unclassified Adinetida | 93331 | 11.3% | unclassified Diplogasterida | 26133 | 5.4% |
| unclassified Rhinosporideacae | 51142 | 6.3% | unclassified Peritrichia II | 42753 | 5.2% | Pfiesteria | 25825 | 5.3% |
| Ephelota | 26003 | 3.2% | unclassified Haplotaxida | 30754 | 3.7% | unclassified LKM11 | 24621 | 5.1% |
| unclassified ConThreeP | 21229 | 2.6% | unclassified Diplogasterida | 27636 | 3.3% | unclassified Adinetida | 22996 | 4.8% |
| Zoothamnium | 20661 | 2.5% | unclassified ConThreeP | 27516 | 3.3% | unclassified Bilateria | 18389 | 3.8% |
| unclassified Rhabditida | 20114 | 2.5% | Ephelota | 19009 | 2.3% | unclassified Haplotaxida | 17974 | 3.7% |
| unclassified Bilateria | 18713 | 2.3% | Zoothamnium | 17836 | 2.2% | unclassified Trebouxiophyceae II | 15767 | 3.3% |
| unclassified Diplogasterida | 18590 | 2.3% | Rhizidiomyces | 16204 | 2.0% | Rhogostoma | 15264 | 3.2% |
| unclassified Ploimida | 16176 | 2.0% | unclassified Podocopida | 15004 | 1.8% | unclassified Rhinosporideacae | 12944 | 2.7% |
| unclassified Haplotaxida | 14599 | 1.8% | unclassified Bilateria | 14559 | 1.8% | Ulva | 9708 | 2.0% |
| Pythium | 14242 | 1.8% | unclassified Ploimida | 10690 | 1.3% | unclassified Stramenopiles | 8755 | 1.8% |
| unclassified Caenogastropoda | 14203 | 1.8% | unclassified Myoida | 8171 | 1.0% | unclassified Podocopida | 7492 | 1.6% |
| Chytridium | 10650 | 1.3% | unclassified Trebouxiophyceae II | 7115 | 0.9% | unclassified Rhabditida | 6974 | 1.4% |
| unclassified Fungi II | 10463 | 1.3% | Rhogostoma | 7032 | 0.8% | unclassified Perkinsidae II | 6953 | 1.4% |
| unclassified Trebouxiophyceae II | 9841 | 1.2% | Chytridium | 6499 | 0.8% | unclassified Intramacronucleata | 6536 | 1.4% |
| unclassified Chytridiomycota II | 8637 | 1.1% | unclassified Bacillariophyceae | 6122 | 0.7% | unclassified Alveolata | 5788 | 1.2% |
| uncultured Choreotrichia | 7109 | 0.9% | unclassified LKM11 | 5456 | 0.7% | unclassified Trebouxiophyceae | 5497 | 1.1% |
| **sum of top 20** | **657470** | **81.1%** | **sum of top 20** | **674667** | **81.4%** | **sum of top 20** | **341728** | **70.7%** |
| total | 810599 | 100.0% | total | 829213 | 100.0% | total | 483071 | 100.0% |

Table S1 continues on page 6.

Table S2 continued from page 5.

| water > 0.2 µm | reads | percent | water > 3.0 µm | reads | percent |
|---|---|---|---|---|---|
| Ostreococcus | 156615 | 22.4% | unclassified ConThreeP | 68193 | 8.1% |
| unclassified Trebouxiophyceae II | 43472 | 6.2% | unclassified Copepoda | 63372 | 7.5% |
| Micromonas | 35637 | 5.1% | Neoceratium | 62110 | 7.3% |
| unclassified Trebouxiophyceae | 30333 | 4.3% | unclassified Diplogasterida | 60845 | 7.2% |
| unclassified MAST-6 | 25435 | 3.6% | unclassified Peritrichia II | 51969 | 6.1% |
| unclassified Syndiniales III | 18883 | 2.7% | unclassified Cercozoa | 39179 | 4.6% |
| unclassified Spongillida | 17241 | 2.5% | unclassified Rhabditida | 36147 | 4.3% |
| Ulva | 16163 | 2.3% | Skeletonema | 32120 | 3.8% |
| Leucocryptos | 14657 | 2.1% | unclassified Adinetida | 27094 | 3.2% |
| Teleaulax | 14535 | 2.1% | Ostreococcus | 25587 | 3.0% |
| unclassified Cercozoa | 14012 | 2.0% | Rhogostoma | 22011 | 2.6% |
| Amoebophrya | 13939 | 2.0% | unclassified Intramacronucleata | 20140 | 2.4% |
| unclassified Stramenopiles | 12412 | 1.8% | unclassified Trebouxiophyceae II | 16843 | 2.0% |
| FV18-2G7 | 12339 | 1.8% | unclassified Cyclopoida | 13512 | 1.6% |
| Ochromonas | 12197 | 1.7% | Thalassiosira | 11675 | 1.4% |
| unclassified Bilateria | 11904 | 1.7% | unclassified Sphaeropleales | 9840 | 1.2% |
| Picomonas | 10058 | 1.4% | unclassified Bilateria | 8672 | 1.0% |
| unclassified Haptoria | 9886 | 1.4% | unclassified Ploimida | 8657 | 1.0% |
| unclassified ConThreeP | 9341 | 1.3% | Scenedesmus | 8343 | 1.0% |
| Pseudopedinella | 9222 | 1.3% | unclassified Calanoida | 7839 | 0.9% |
| **sum of top 20** | **488281** | **69.9%** | **sum of top 20** | **594148** | **70.3%** |
| total | 698248 | 100.0% | total | 845688 | 100.0% |

**Fig. S2.** Phylogenetic trees of representative sequences of the 20 most abundant taxa (in bold and with rank/position in top 20 list, see also Table S2) and their close relatives for each substrate type. Scale bar represents 10 nucleotide substitutions per 100 nucleotides. GenBank accession numbers and bootstrap values are displayed.

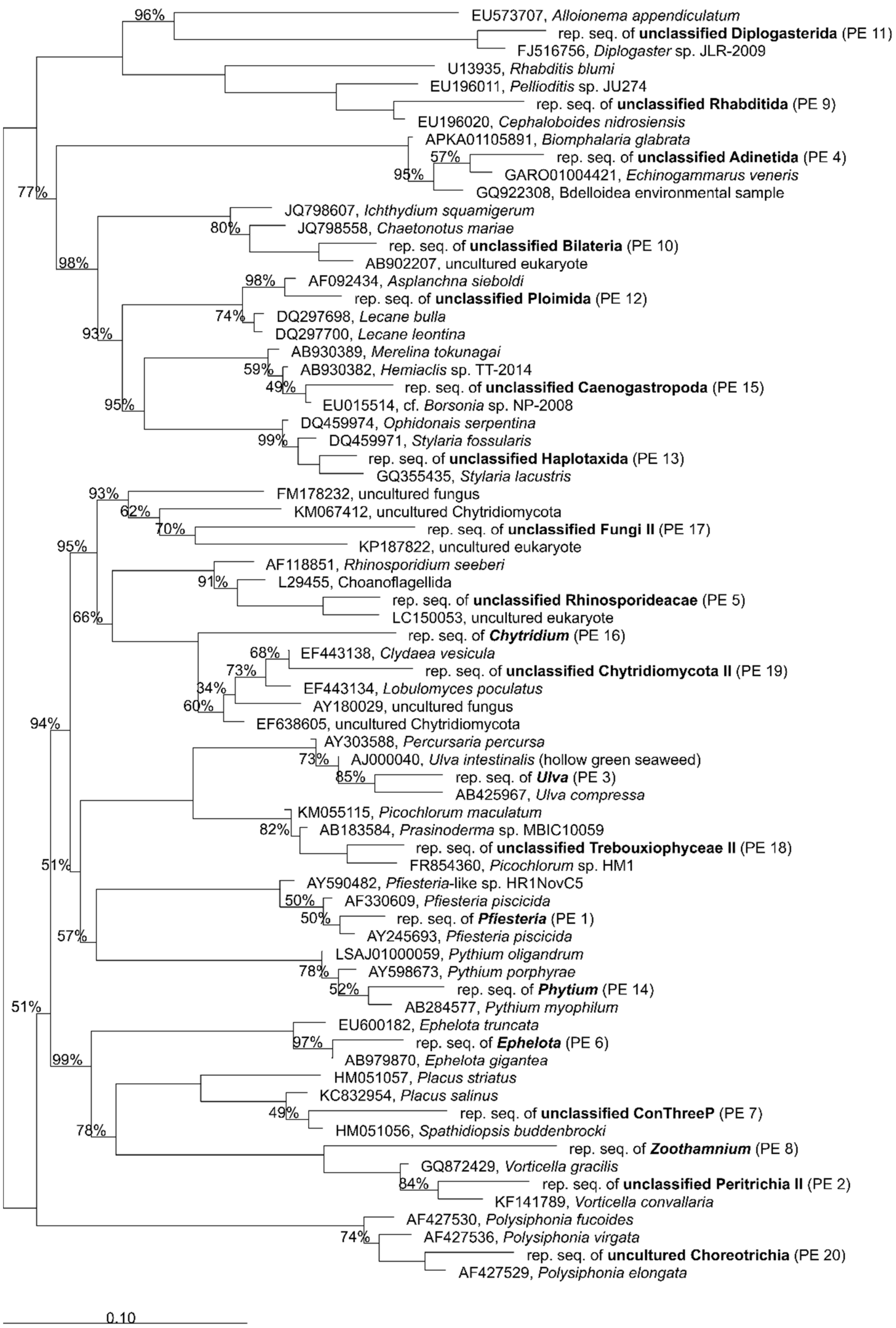

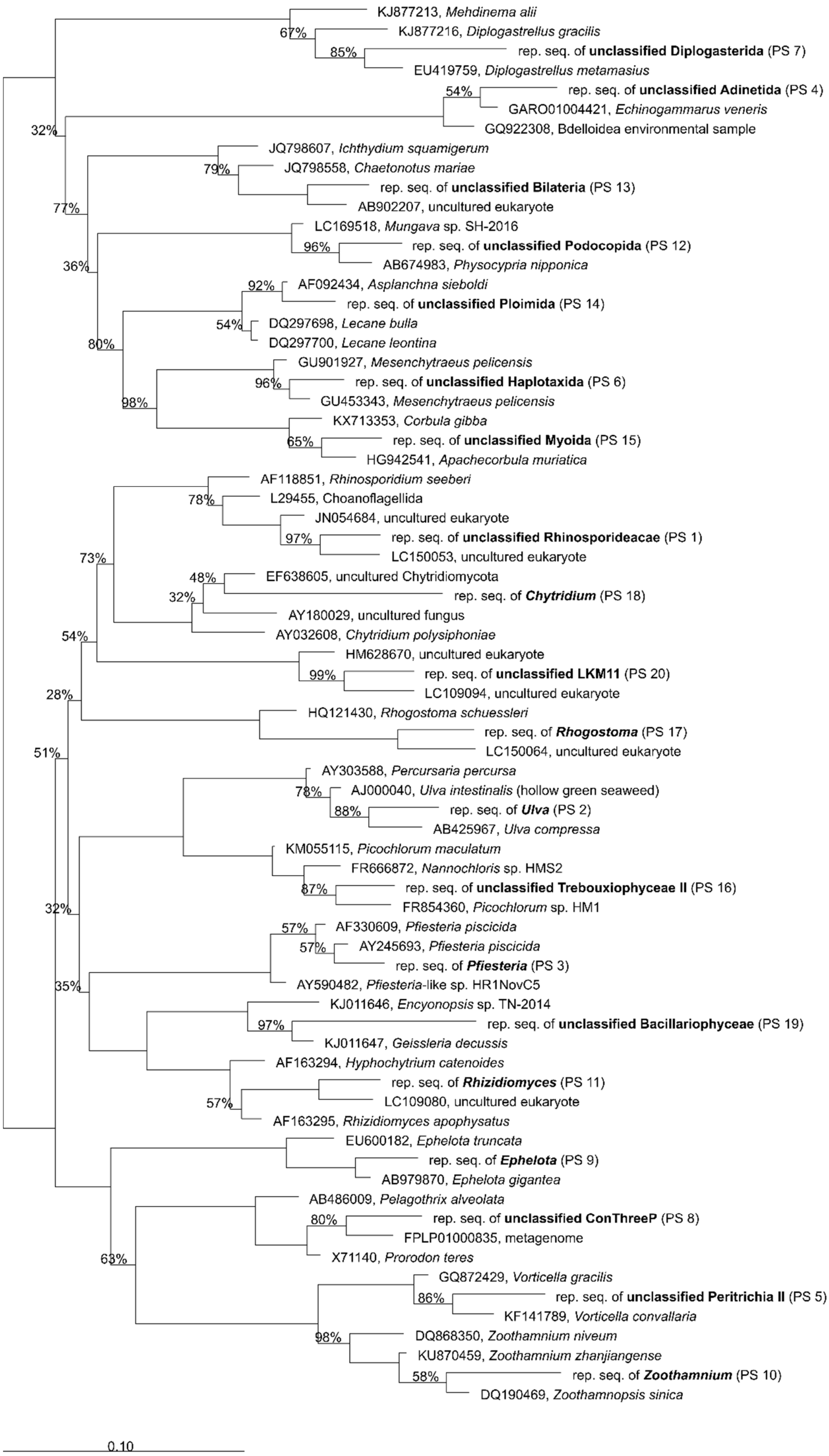
KJ877213, *Mehdinema alii*
67%
KJ877216, *Diplogastrellus gracilis*
85%
rep. seq. of **unclassified Diplogasterida** (PS 7)
EU419759, *Diplogastrellus metamasius*
54%
rep. seq. of **unclassified Adinetida** (PS 4)
GARO01004421, *Echinogammarus veneris*
32%
GQ922308, Bdelloidea environmental sample
JQ798607, *Ichthydium squamigerum*
79%
JQ798558, *Chaetonotus mariae*
rep. seq. of **unclassified Bilateria** (PS 13)
77%
AB902207, uncultured eukaryote
LC169518, *Mungava* sp. SH-2016
96%
rep. seq. of **unclassified Podocopida** (PS 12)
36%
AB674983, *Physocypria nipponica*
92%
AF092434, *Asplanchna sieboldi*
rep. seq. of **unclassified Ploimida** (PS 14)
54%
DQ297698, *Lecane bulla*
80%
DQ297700, *Lecane leontina*
GU901927, *Mesenchytraeus pelicensis*
96%
rep. seq. of **unclassified Haplotaxida** (PS 6)
98%
GU453343, *Mesenchytraeus pelicensis*
KX713353, *Corbula gibba*
65%
rep. seq. of **unclassified Myoida** (PS 15)
HG942541, *Apachecorbula muriatica*
AF118851, *Rhinosporidium seeberi*
78%
L29455, Choanoflagellida
JN054684, uncultured eukaryote
97%
rep. seq. of **unclassified Rhinosporideacae** (PS 1)
73%
LC150053, uncultured eukaryote
48%
EF638605, uncultured Chytridiomycota
32%
rep. seq. of ***Chytridium*** (PS 18)
AY180029, uncultured fungus
54%
AY032608, *Chytridium polysiphoniae*
HM628670, uncultured eukaryote
99%
rep. seq. of **unclassified LKM11** (PS 20)
28%
LC109094, uncultured eukaryote
HQ121430, *Rhogostoma schuessleri*
rep. seq. of ***Rhogostoma*** (PS 17)
51%
LC150064, uncultured eukaryote
AY303588, *Percursaria percursa*
78%
AJ000040, *Ulva intestinalis* (hollow green seaweed)
88%
rep. seq. of ***Ulva*** (PS 2)
AB425967, *Ulva compressa*
KM055115, *Picochlorum maculatum*
FR666872, *Nannochloris* sp. HMS2
87%
rep. seq. of **unclassified Trebouxiophyceae II** (PS 16)
32%
FR854360, *Picochlorum* sp. HM1
57%
AF330609, *Pfiesteria piscicida*
57%
AY245693, *Pfiesteria piscicida*
rep. seq. of ***Pfiesteria*** (PS 3)
35%
AY590482, *Pfiesteria*-like sp. HR1NovC5
KJ011646, *Encyonopsis* sp. TN-2014
97%
rep. seq. of **unclassified Bacillariophyceae** (PS 19)
KJ011647, *Geissleria decussis*
AF163294, *Hyphochytrium catenoides*
rep. seq. of ***Rhizidiomyces*** (PS 11)
57%
LC109080, uncultured eukaryote
AF163295, *Rhizidiomyces apophysatus*
EU600182, *Ephelota truncata*
rep. seq. of ***Ephelota*** (PS 9)
AB979870, *Ephelota gigantea*
AB486009, *Pelagothrix alveolata*
80%
rep. seq. of **unclassified ConThreeP** (PS 8)
FPLP01000835, metagenome
63%
X71140, *Prorodon teres*
GQ872429, *Vorticella gracilis*
86%
rep. seq. of **unclassified Peritrichia II** (PS 5)
KF141789, *Vorticella convallaria*
98%
DQ868350, *Zoothamnium niveum*
KU870459, *Zoothamnium zhanjiangense*
58%
rep. seq. of ***Zoothamnium*** (PS 10)
DQ190469, *Zoothamnopsis sinica*
0.10

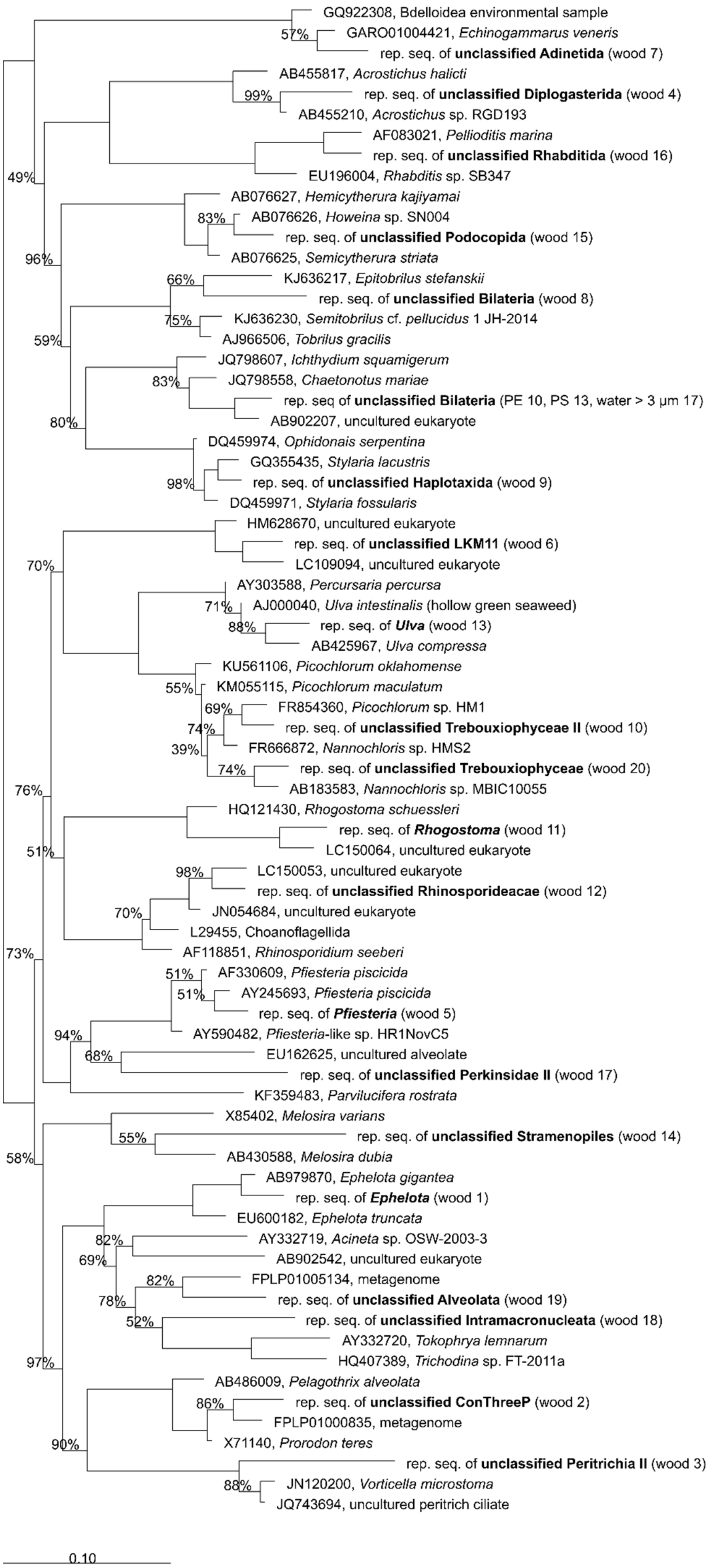
GQ922308, Bdelloidea environmental sample
57%
GARO01004421, Echinogammarus veneris
rep. seq. of unclassified Adinetida (wood 7)
AB455817, Acrostichus halicti
99%
rep. seq. of unclassified Diplogasterida (wood 4)
AB455210, Acrostichus sp. RGD193
AF083021, Pellioditis marina
rep. seq. of unclassified Rhabditida (wood 16)
49%
EU196004, Rhabditis sp. SB347
AB076627, Hemicytherura kajiyamai
83%
AB076626, Howeina sp. SN004
rep. seq. of unclassified Podocopida (wood 15)
96%
AB076625, Semicytherura striata
66%
KJ636217, Epitobrilus stefanskii
rep. seq. of unclassified Bilateria (wood 8)
75%
KJ636230, Semitobrilus cf. pellucidus 1 JH-2014
59%
AJ966506, Tobrilus gracilis
JQ798607, Ichthydium squamigerum
83%
JQ798558, Chaetonotus mariae
rep. seq of unclassified Bilateria (PE 10, PS 13, water > 3 µm 17)
80%
AB902207, uncultured eukaryote
DQ459974, Ophidonais serpentina
GQ355435, Stylaria lacustris
98%
rep. seq. of unclassified Haplotaxida (wood 9)
DQ459971, Stylaria fossularis
HM628670, uncultured eukaryote
rep. seq. of unclassified LKM11 (wood 6)
70%
LC109094, uncultured eukaryote
AY303588, Percursaria percursa
71%
AJ000040, Ulva intestinalis (hollow green seaweed)
88%
rep. seq. of Ulva (wood 13)
AB425967, Ulva compressa
KU561106, Picochlorum oklahomense
55%
KM055115, Picochlorum maculatum
69%
FR854360, Picochlorum sp. HM1
74%
rep. seq. of unclassified Trebouxiophyceae II (wood 10)
39%
FR666872, Nannochloris sp. HMS2
74%
rep. seq. of unclassified Trebouxiophyceae (wood 20)
76%
AB183583, Nannochloris sp. MBIC10055
HQ121430, Rhogostoma schuessleri
rep. seq. of Rhogostoma (wood 11)
51%
LC150064, uncultured eukaryote
98%
LC150053, uncultured eukaryote
rep. seq. of unclassified Rhinosporideacae (wood 12)
70%
JN054684, uncultured eukaryote
L29455, Choanoflagellida
73%
AF118851, Rhinosporidium seeberi
51%
AF330609, Pfiesteria piscicida
51%
AY245693, Pfiesteria piscicida
rep. seq. of Pfiesteria (wood 5)
94%
AY590482, Pfiesteria-like sp. HR1NovC5
68%
EU162625, uncultured alveolate
rep. seq. of unclassified Perkinsidae II (wood 17)
KF359483, Parvilucifera rostrata
X85402, Melosira varians
55%
rep. seq. of unclassified Stramenopiles (wood 14)
58%
AB430588, Melosira dubia
AB979870, Ephelota gigantea
rep. seq. of Ephelota (wood 1)
EU600182, Ephelota truncata
82%
AY332719, Acineta sp. OSW-2003-3
69%
AB902542, uncultured eukaryote
82%
FPLP01005134, metagenome
78%
rep. seq. of unclassified Alveolata (wood 19)
52%
rep. seq. of unclassified Intramacronucleata (wood 18)
AY332720, Tokophrya lemnarum
97%
HQ407389, Trichodina sp. FT-2011a
AB486009, Pelagothrix alveolata
86%
rep. seq. of unclassified ConThreeP (wood 2)
FPLP01000835, metagenome
90%
X71140, Prorodon teres
rep. seq. of unclassified Peritrichia II (wood 3)
88%
JN120200, Vorticella microstoma
JQ743694, uncultured peritrich ciliate
0.10

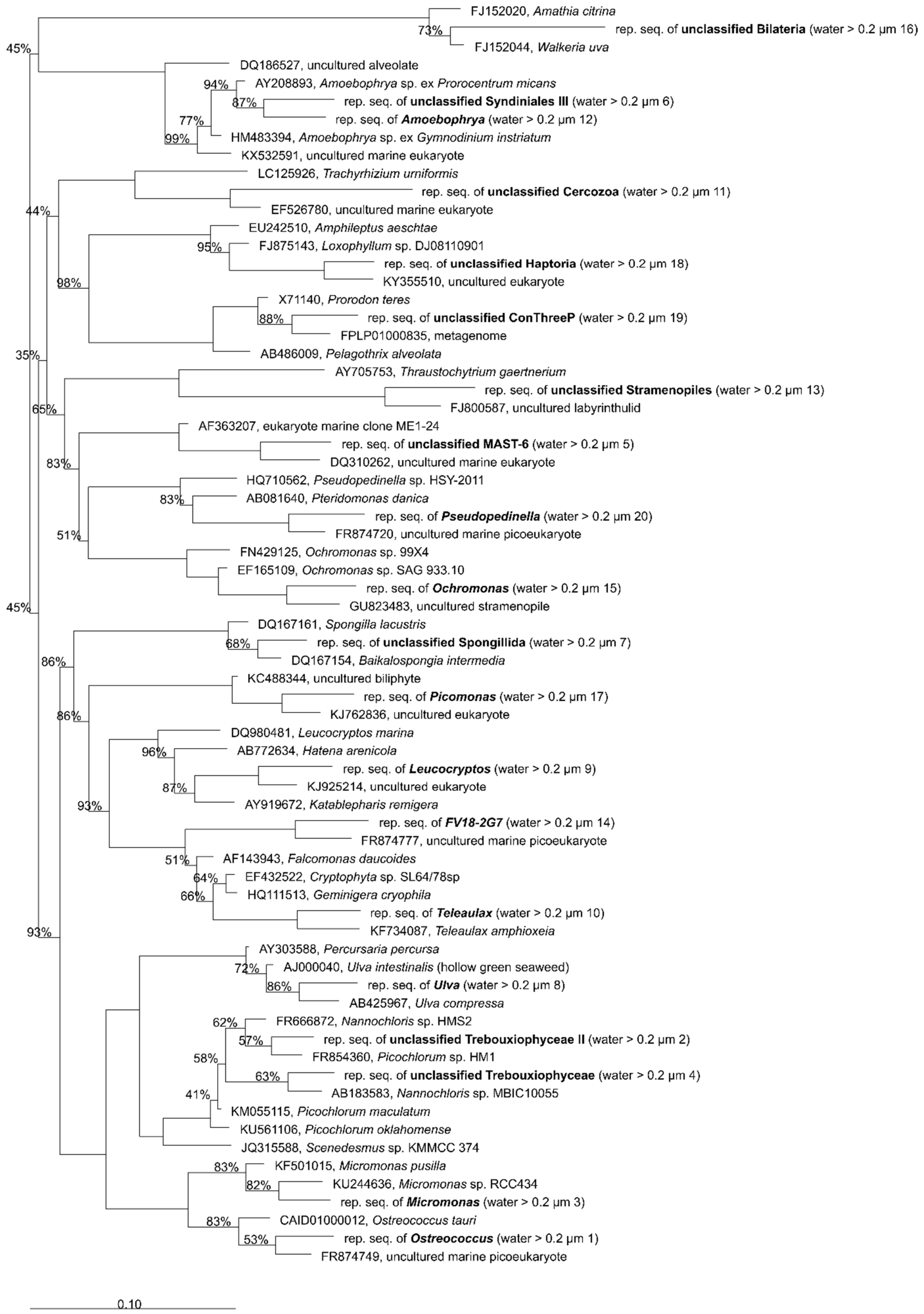

FJ152020, Amathia citrina
rep. seq. of unclassified Bilateria (water > 0.2 µm 16)
FJ152044, Walkeria uva
DQ186527, uncultured alveolate
AY208893, Amoebophrya sp. ex Prorocentrum micans
rep. seq. of unclassified Syndiniales III (water > 0.2 µm 6)
rep. seq. of Amoebophrya (water > 0.2 µm 12)
HM483394, Amoebophrya sp. ex Gymnodinium instriatum
KX532591, uncultured marine eukaryote
LC125926, Trachyrhizium urniformis
rep. seq. of unclassified Cercozoa (water > 0.2 µm 11)
EF526780, uncultured marine eukaryote
EU242510, Amphileptus aeschtae
FJ875143, Loxophyllum sp. DJ08110901
rep. seq. of unclassified Haptoria (water > 0.2 µm 18)
KY355510, uncultured eukaryote
X71140, Prorodon teres
rep. seq. of unclassified ConThreeP (water > 0.2 µm 19)
FPLP01000835, metagenome
AB486009, Pelagothrix alveolata
AY705753, Thraustochytrium gaertnerium
rep. seq. of unclassified Stramenopiles (water > 0.2 µm 13)
FJ800587, uncultured labyrinthulid
AF363207, eukaryote marine clone ME1-24
rep. seq. of unclassified MAST-6 (water > 0.2 µm 5)
DQ310262, uncultured marine eukaryote
HQ710562, Pseudopedinella sp. HSY-2011
AB081640, Pteridomonas danica
rep. seq. of Pseudopedinella (water > 0.2 µm 20)
FR874720, uncultured marine picoeukaryote
FN429125, Ochromonas sp. 99X4
EF165109, Ochromonas sp. SAG 933.10
rep. seq. of Ochromonas (water > 0.2 µm 15)
GU823483, uncultured stramenopile
DQ167161, Spongilla lacustris
rep. seq. of unclassified Spongillida (water > 0.2 µm 7)
DQ167154, Baikalospongia intermedia
KC488344, uncultured biliphyte
rep. seq. of Picomonas (water > 0.2 µm 17)
KJ762836, uncultured eukaryote
DQ980481, Leucocryptos marina
AB772634, Hatena arenicola
rep. seq. of Leucocryptos (water > 0.2 µm 9)
KJ925214, uncultured eukaryote
AY919672, Katablepharis remigera
rep. seq. of FV18-2G7 (water > 0.2 µm 14)
FR874777, uncultured marine picoeukaryote
AF143943, Falcomonas daucoides
EF432522, Cryptophyta sp. SL64/78sp
HQ111513, Geminigera cryophila
rep. seq. of Teleaulax (water > 0.2 µm 10)
KF734087, Teleaulax amphioxeia
AY303588, Percursaria percursa
AJ000040, Ulva intestinalis (hollow green seaweed)
rep. seq. of Ulva (water > 0.2 µm 8)
AB425967, Ulva compressa
FR666872, Nannochloris sp. HMS2
rep. seq. of unclassified Trebouxiophyceae II (water > 0.2 µm 2)
FR854360, Picochlorum sp. HM1
rep. seq. of unclassified Trebouxiophyceae (water > 0.2 µm 4)
AB183583, Nannochloris sp. MBIC10055
KM055115, Picochlorum maculatum
KU561106, Picochlorum oklahomense
JQ315588, Scenedesmus sp. KMMCC 374
KF501015, Micromonas pusilla
KU244636, Micromonas sp. RCC434
rep. seq. of Micromonas (water > 0.2 µm 3)
CAID01000012, Ostreococcus tauri
rep. seq. of Ostreococcus (water > 0.2 µm 1)
FR874749, uncultured marine picoeukaryote
45%
73%
94%
87%
77%
99%
44%
95%
98%
88%
35%
65%
83%
83%
51%
45%
68%
86%
86%
96%
87%
93%
51%
64%
66%
93%
72%
86%
62%
57%
58%
63%
41%
83%
82%
83%
53%
0.10

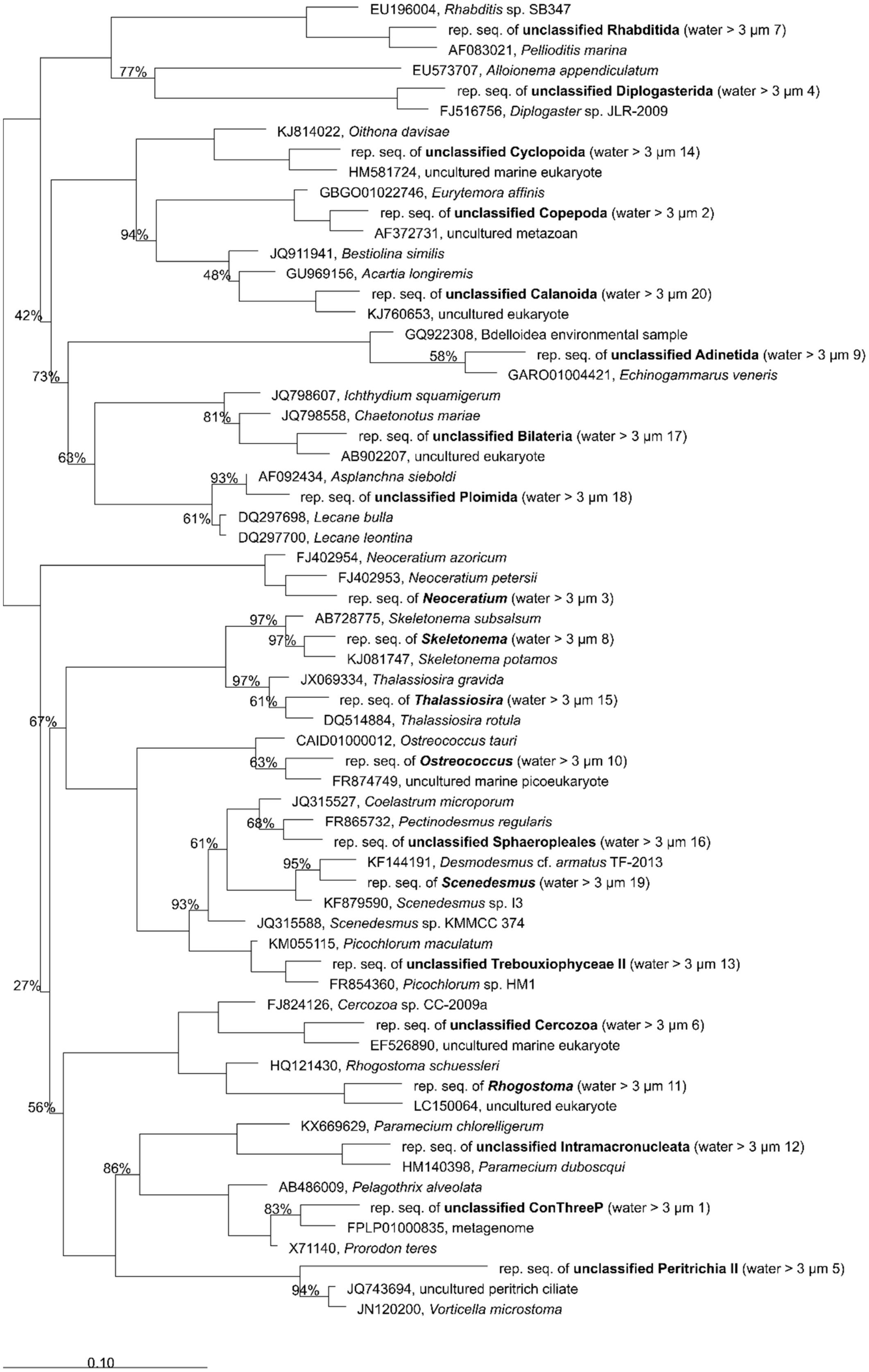
EU196004, Rhabditis sp. SB347
rep. seq. of unclassified Rhabditida (water > 3 µm 7)
AF083021, Pellioditis marina
77%
EU573707, Alloionema appendiculatum
rep. seq. of unclassified Diplogasterida (water > 3 µm 4)
FJ516756, Diplogaster sp. JLR-2009
KJ814022, Oithona davisae
rep. seq. of unclassified Cyclopoida (water > 3 µm 14)
HM581724, uncultured marine eukaryote
GBGO01022746, Eurytemora affinis
rep. seq. of unclassified Copepoda (water > 3 µm 2)
94%
AF372731, uncultured metazoan
JQ911941, Bestiolina similis
48%
GU969156, Acartia longiremis
rep. seq. of unclassified Calanoida (water > 3 µm 20)
42%
KJ760653, uncultured eukaryote
GQ922308, Bdelloidea environmental sample
58%
rep. seq. of unclassified Adinetida (water > 3 µm 9)
73%
GARO01004421, Echinogammarus veneris
JQ798607, Ichthydium squamigerum
81%
JQ798558, Chaetonotus mariae
rep. seq. of unclassified Bilateria (water > 3 µm 17)
63%
AB902207, uncultured eukaryote
93%
AF092434, Asplanchna sieboldi
rep. seq. of unclassified Ploimida (water > 3 µm 18)
61%
DQ297698, Lecane bulla
DQ297700, Lecane leontina
FJ402954, Neoceratium azoricum
FJ402953, Neoceratium petersii
rep. seq. of Neoceratium (water > 3 µm 3)
97%
AB728775, Skeletonema subsalsum
97%
rep. seq. of Skeletonema (water > 3 µm 8)
KJ081747, Skeletonema potamos
97%
JX069334, Thalassiosira gravida
61%
rep. seq. of Thalassiosira (water > 3 µm 15)
67%
DQ514884, Thalassiosira rotula
CAID01000012, Ostreococcus tauri
63%
rep. seq. of Ostreococcus (water > 3 µm 10)
FR874749, uncultured marine picoeukaryote
JQ315527, Coelastrum microporum
68%
FR865732, Pectinodesmus regularis
61%
rep. seq. of unclassified Sphaeropleales (water > 3 µm 16)
95%
KF144191, Desmodesmus cf. armatus TF-2013
rep. seq. of Scenedesmus (water > 3 µm 19)
93%
KF879590, Scenedesmus sp. I3
JQ315588, Scenedesmus sp. KMMCC 374
KM055115, Picochlorum maculatum
rep. seq. of unclassified Trebouxiophyceae II (water > 3 µm 13)
27%
FR854360, Picochlorum sp. HM1
FJ824126, Cercozoa sp. CC-2009a
rep. seq. of unclassified Cercozoa (water > 3 µm 6)
EF526890, uncultured marine eukaryote
HQ121430, Rhogostoma schuessleri
rep. seq. of Rhogostoma (water > 3 µm 11)
56%
LC150064, uncultured eukaryote
KX669629, Paramecium chlorelligerum
rep. seq. of unclassified Intramacronucleata (water > 3 µm 12)
86%
HM140398, Paramecium duboscqui
AB486009, Pelagothrix alveolata
83%
rep. seq. of unclassified ConThreeP (water > 3 µm 1)
FPLP01000835, metagenome
X71140, Prorodon teres
rep. seq. of unclassified Peritrichia II (water > 3 µm 5)
94%
JQ743694, uncultured peritrich ciliate
JN120200, Vorticella microstoma
0.10

**Table S3.** Results of permutational multivariate analysis of variance (PERMANOVA) after 999 permutations for the factors substrate and location as well as their interaction term. df = degrees of freedom, Sq = squares

| **PERMANOVA (999 permutations)** | | | | | | |
|---|---|---|---|---|---|---|
| | df | sum Sq | mean Sq | F-Model | **$R^2$** | **p-value** |
| substrate | 4 | 3.614 | 0.903 | 16.715 | **0.140** | **0.001** |
| location | 6 | 12.030 | 2.005 | 37.095 | **0.467** | **0.001** |
| substrate:location | 24 | 6.876 | 0.286 | 5.301 | **0.267** | **0.001** |
| Residuals | 60 | 3.243 | 0.054 | | 0.126 | |
| Total | 94 | 25.763 | | | 100.0 | |

**Table S4.** Results of permutation tests for homogeneity of multivariate dispersions for different substrate types and locations. P-values over 0.05 indicate a homogenous dispersion. df = degrees of freedom, Sq = squares

| **Dispersion levels for substrates types** | | | | |
|---|---|---|---|---|
| Average distance to median: | | | | |
| PE | PS | wood | water > 0.2 µm | water > 3.0 µm |
| 0.490 | 0.498 | 0.478 | 0.448 | 0.462 |

| Permutation test for homogeneity of multivariate dispersions (999 permutations): | | | | | |
|---|---|---|---|---|---|
| | df | sum Sq | mean Sq | F-Model | **p-value** |
| Groups | 4 | 0.032 | 0.008 | 0.961 | 0.454 |
| Residuals | 90 | 0.749 | 0.008 | | |

| **Dispersion levels for locations** | | | | | | |
|---|---|---|---|---|---|---|
| Average distance to median: | | | | | | |
| Station 1 | Station 2 | Station 3 | Station 4 | Station 5 | Station 6 | Station 7 |
| 0.407 | 0.365 | 0.415 | 0.377 | 0.383 | 0.324 | 0.311 |

| Permutation test for homogeneity of multivariate dispersions (999 permutations): | | | | | |
|---|---|---|---|---|---|
| | df | sum Sq | mean Sq | F-Model | **p-value** |
| Groups | 6 | 0.131 | 0.022 | 1.858 | 0.110 |
| Residuals | 88 | 1.036 | 0.012 | | |

**Table S5.** Results of pairwise PERMANOVA (999 permutations) and Bray-Curtis (BC) similarity for different substrate types and locations. P-adjustment according to Benjamini and Hochberg, 1995.

| **Pairwise PERMANOVA for different substrate types** | | | | | |
|---|---|---|---|---|---|
| **pairs** | **F-Model** | **$R^2$** | **p-value** | **p adjusted** | **BC similarity** |
| PE vs PS | 0.418 | 0.010 | 0.942 | 0.942 | 78.7% |
| PE vs wood | 2.157 | 0.051 | 0.028 | **0.035** | 68.1% |
| PE vs water>0.2µm | 6.455 | 0.145 | 0.001 | **0.003** | 42.1% |
| PE vs water>3.0µm | 3.080 | 0.088 | 0.006 | **0.012** | 54.8% |
| PS vs wood | 2.185 | 0.052 | 0.035 | **0.039** | 69.5% |
| PS vs water>0.2µm | 6.764 | 0.151 | 0.001 | **0.003** | 41.8% |
| PS vs water>3.0µm | 3.506 | 0.099 | 0.004 | **0.010** | 53.7% |
| wood vs water>0.2µm | 6.107 | 0.138 | 0.001 | **0.003** | 46.2% |
| wood vs water>3.0µm | 2.728 | 0.079 | 0.015 | **0.021** | 59.4% |
| water>0.2µm vs water>3.0µm | 3.828 | 0.113 | 0.013 | **0.021** | 56.2% |
| **Pairwise PERMANOVA for different locations** | | | | | |
| Station 1 vs Station 2 | 5.895 | 0.197 | 0.001 | **0.001** | 54.3% |
| Station 1 vs Station 3 | 4.422 | 0.156 | 0.001 | **0.001** | 56.4% |
| Station 1 vs Station 4 | 7.936 | 0.249 | 0.001 | **0.001** | 46.0% |
| Station 1 vs Station 5 | 9.547 | 0.285 | 0.001 | **0.001** | 43.0% |
| Station 1 vs Station 6 | 20.657 | 0.443 | 0.001 | **0.001** | 20.7% |
| Station 1 vs Station 7 | 19.091 | 0.423 | 0.001 | **0.001** | 24.7% |
| Station 2 vs Station 3 | 3.693 | 0.133 | 0.005 | **0.005** | 65.5% |
| Station 2 vs Station 4 | 7.866 | 0.247 | 0.001 | **0.001** | 48.8% |
| Station 2 vs Station 5 | 10.631 | 0.307 | 0.001 | **0.001** | 43.3% |
| Station 2 vs Station 6 | 21.691 | 0.455 | 0.001 | **0.001** | 24.8% |
| Station 2 vs Station 7 | 21.345 | 0.451 | 0.001 | **0.001** | 28.0% |
| Station 3 vs Station 4 | 8.106 | 0.252 | 0.001 | **0.001** | 42.1% |
| Station 3 vs Station 5 | 11.190 | 0.318 | 0.001 | **0.001** | 39.4% |
| Station 3 vs Station 6 | 18.635 | 0.418 | 0.001 | **0.001** | 24.6% |
| Station 3 vs Station 7 | 18.012 | 0.409 | 0.001 | **0.001** | 25.7% |
| Station 4 vs Station 5 | 4.360 | 0.154 | 0.003 | **0.003** | 70.4% |
| Station 4 vs Station 6 | 21.961 | 0.458 | 0.001 | **0.001** | 27.3% |
| Station 4 vs Station 7 | 23.208 | 0.472 | 0.001 | **0.001** | 27.9% |
| Station 5 vs Station 6 | 18.550 | 0.416 | 0.001 | **0.001** | 29.2% |
| Station 5 vs Station 7 | 19.253 | 0.425 | 0.001 | **0.001** | 30.3% |
| Station 6 vs Station 7 | 6.198 | 0.181 | 0.001 | **0.001** | 66.1% |

**Table S6.** Results of indicator species analysis with taxa associated to each substrate type. For each taxon the corresponding phylum, kingdom, indicator value and adjusted p-value (according to Benjamini and Hochberg, 1995) are given. Taxonomic information is based on SILVA (Yilmaz et al., 2014) database v128.

| **Eukaryotic taxa associated to substrate types** | | | | |
|---|---|---|---|---|
| **taxon** | **phylum** | **kingdom** | **indicator value** | **p-value adjusted** |
| **PE (polyethylene)** | | | | |
| unclassified Monogononta | Rotifera | Metazoa (Animalia) | 0.549 | 0.033 |
| **PS (polystyrene)** | | | | |
| unclassified Ulvophyceae II | Chlorophyta (unclassified phylum) | Chloroplastida | 0.547 | 0.025 |
| **wood** | | | | |
| Candida | Ascomycota | Fungi | 0.843 | 0.005 |
| Ogataea | Ascomycota | Fungi | 0.787 | 0.005 |
| Kuraishia | Ascomycota | Fungi | 0.724 | 0.005 |
| unclassified Saccharomycetales III | Ascomycota | Fungi | 0.669 | 0.005 |
| unclassified Cyrtophoria | Ciliophora | Alveolata | 0.630 | 0.007 |
| Pseudocohnilembus | Ciliophora | Alveolata | 0.618 | 0.005 |
| Wickerhamomyces | Ascomycota | Fungi | 0.588 | 0.007 |
| Choricystis | Chlorophyta (unclassified phylum) | Chloroplastida | 0.585 | 0.019 |
| Saccharomycopsis | Ascomycota | Fungi | 0.577 | 0.005 |
| Pichia | Ascomycota | Fungi | 0.558 | 0.010 |
| Clavispora | Ascomycota | Fungi | 0.524 | 0.017 |
| unclassified Cystofilobasidiaceae II | Basidiomycota | Fungi | 0.522 | 0.010 |
| Condylostoma | Ciliophora | Alveolata | 0.470 | 0.050 |
| **water > 0.2 µm** | | | | |
| Ochromonas | Ochrophyta | Stramenopiles | 0.797 | 0.005 |
| E222 | Ochrophyta | Stramenopiles | 0.786 | 0.005 |
| Paraphysomonas | Ochrophyta | Stramenopiles | 0.779 | 0.005 |
| unclassified MAST-4D | MAST-4 | Stramenopiles | 0.764 | 0.005 |

| | | | | |
|---|---|---|---|---|
| unclassified Pedinellales II | Ochrophyta | Stramenopiles | 0.750 | 0.005 |
| Bolidomonas | Ochrophyta | Stramenopiles | 0.746 | 0.005 |
| unclassified DH147-EKD10 | DH147-EKD10 | Eukaryota (unclassified kingdom) | 0.745 | 0.005 |
| unclassified Chrysophyceae | Ochrophyta | Stramenopiles | 0.734 | 0.005 |
| unclassified MAST-2 | MAST-2 | Stramenopiles | 0.723 | 0.005 |
| unclassified Mamiellophyceae | Chlorophyta (unclassified phylum) | Chloroplastida | 0.711 | 0.005 |
| Chrysochromulina | Prymnesiophyceae | Haptophyta | 0.708 | 0.005 |
| Picomonas | Picozoa | Eukaryota (unclassified kingdom) | 0.707 | 0.005 |
| Mamiella | Chlorophyta (unclassified phylum) | Chloroplastida | 0.705 | 0.005 |
| Micromonas | Chlorophyta (unclassified phylum) | Chloroplastida | 0.704 | 0.005 |
| unclassified Cryptomonadales | Cryptomonadales | Cryptophyceae | 0.704 | 0.005 |
| Ostreococcus | Chlorophyta (unclassified phylum) | Chloroplastida | 0.704 | 0.005 |
| Geminigera | Cryptomonadales | Cryptophyceae | 0.701 | 0.005 |
| Paragymnodinium | Dinoflagellata | Alveolata | 0.697 | 0.005 |
| unclassified Prymnesiales | Prymnesiophyceae | Haptophyta | 0.696 | 0.005 |
| Hemiselmis | Cryptomonadales | Cryptophyceae | 0.693 | 0.005 |
| unclassified Mamiellales | Chlorophyta (unclassified phylum) | Chloroplastida | 0.681 | 0.005 |
| unclassified Syndiniales Group I | Protalveolata | Alveolata | 0.679 | 0.005 |
| unclassified Chromulinales | Ochrophyta | Stramenopiles | 0.677 | 0.005 |
| Teleaulax | Cryptomonadales | Cryptophyceae | 0.668 | 0.005 |
| uncultured Cryptomonadales | Cryptomonadales | Cryptophyceae | 0.668 | 0.005 |
| Spumella | Ochrophyta | Stramenopiles | 0.665 | 0.005 |
| unclassified LG21-05 | Ochrophyta | Stramenopiles | 0.664 | 0.005 |
| unclassified Chloroplastida | Chloroplastida (unclassified phylum) | Chloroplastida | 0.653 | 0.005 |
| unclassified A31 | Protalveolata | Alveolata | 0.650 | 0.005 |
| unclassified Dictyochophyceae | Ochrophyta | Stramenopiles | 0.650 | 0.005 |
| Amoebophrya | Protalveolata | Alveolata | 0.650 | 0.007 |
| unclassified Syndiniales III | Protalveolata | Alveolata | 0.645 | 0.007 |
| Leucocryptos | Kathablepharidae | Cryptophyceae | 0.641 | 0.005 |
| unclassified Spongillida | Porifera | Metazoa (Animalia) | 0.641 | 0.005 |
| uncultured Oligotrichia | Ciliophora | Alveolata | 0.639 | 0.007 |

| | | | | |
|---|---|---|---|---|
| FV18-2G7 | Cryptomonadales | Cryptophyceae | 0.636 | 0.005 |
| Prasinoderma | Chlorophyta (unclassified phylum) | Chloroplastida | 0.632 | 0.005 |
| Tiarina | Ciliophora | Alveolata | 0.623 | 0.005 |
| Uroglena | Ochrophyta | Stramenopiles | 0.621 | 0.005 |
| unclassified Cryptomonadales II | Cryptomonadales | Cryptophyceae | 0.618 | 0.005 |
| unclassified MAST-6 | MAST-6 | Stramenopiles | 0.618 | 0.007 |
| unclassified OLI11255 | OLI11255 | Alveolata | 0.614 | 0.007 |
| unclassified Exobasidiales | Basidiomycota | Fungi | 0.609 | 0.005 |
| unclassified Anthoathecata | Cnidaria | Metazoa (Animalia) | 0.605 | 0.010 |
| Chaetoceros | Ochrophyta | Stramenopiles | 0.602 | 0.010 |
| Pseudopedinella | Ochrophyta | Stramenopiles | 0.600 | 0.007 |
| unclassified Chlorophyta | Chlorophyta (unclassified phylum) | Chloroplastida | 0.598 | 0.005 |
| Gyrodinium | Dinoflagellata | Alveolata | 0.591 | 0.007 |
| unclassified Choreotrichia | Ciliophora | Alveolata | 0.588 | 0.007 |
| Diaphanoeca | Choanoflagellida | Holozoa (unclassified kingdom) | 0.579 | 0.005 |
| unclassified MAST-3F | MAST-3 | Stramenopiles | 0.577 | 0.007 |
| unclassified MAST-3E | MAST-3 | Stramenopiles | 0.576 | 0.007 |
| unclassified Pedinellales | Ochrophyta | Stramenopiles | 0.574 | 0.013 |
| Prymnesium | Prymnesiophyceae | Haptophyta | 0.568 | 0.010 |
| unclassified Ctenostomatida | Bryozoa | Metazoa (Animalia) | 0.559 | 0.015 |
| Pseudochattonella | Ochrophyta | Stramenopiles | 0.556 | 0.005 |
| unclassified MAST-12A | MAST-12 | Stramenopiles | 0.553 | 0.005 |
| unclassified Oligotrichia | Ciliophora | Alveolata | 0.552 | 0.027 |
| unclassified Hydrozoa | Cnidaria | Metazoa (Animalia) | 0.549 | 0.010 |
| Vermamoeba | Tubulinea | Amoebozoa | 0.546 | 0.007 |
| SS1-E01-69 | Ochrophyta | Stramenopiles | 0.543 | 0.005 |
| Pirsonia | Cercozoa | Rhizaria | 0.524 | 0.038 |
| Pycnococcus | Chlorophyta (unclassified phylum) | Chloroplastida | 0.523 | 0.005 |
| unclassified P34-45 | Ochrophyta | Stramenopiles | 0.519 | 0.015 |
| unclassified Sarcinochrysidales | Ochrophyta | Stramenopiles | 0.518 | 0.005 |
| Imantonia | Prymnesiophyceae | Haptophyta | 0.513 | 0.005 |

| | | | | |
|---|---|---|---|---|
| unclassified Phyllodocida | Annelida | Metazoa (Animalia) | 0.505 | 0.017 |
| unclassified LG01-09 | Ochrophyta | Stramenopiles | 0.494 | 0.019 |
| OLI16029 | Prymnesiophyceae | Haptophyta | 0.482 | 0.007 |
| unclassified Prasinophytae II | Chlorophyta (unclassified phylum) | Chloroplastida | 0.466 | 0.044 |
| Poterioochromonas | Ochrophyta | Stramenopiles | 0.463 | 0.023 |
| Gymnophrys | Cercozoa | Rhizaria | 0.437 | 0.048 |
| Phaeocystis | Prymnesiophyceae | Haptophyta | 0.424 | 0.015 |
| unclassified Syndiniales Group III | Protalveolata | Alveolata | 0.423 | 0.019 |
| Sarcinochrysis | Ochrophyta | Stramenopiles | 0.402 | 0.044 |
| Dictyocha | Ochrophyta | Stramenopiles | 0.354 | 0.038 |
| **water > 3.0 µm** | | | | |
| Trachydiscus | Ochrophyta | Stramenopiles | 0.743 | 0.005 |
| Aphanomyces | Peronosporomycetes | Stramenopiles | 0.728 | 0.005 |
| Pteromonas | Chlorophyta (unclassified phylum) | Chloroplastida | 0.728 | 0.005 |
| unclassified Conoidasida | Apicomplexa | Alveolata | 0.714 | 0.005 |
| Hydrodictyon | Chlorophyta (unclassified phylum) | Chloroplastida | 0.695 | 0.005 |
| Discostella | Ochrophyta | Stramenopiles | 0.685 | 0.005 |
| Oocystis | Chlorophyta (unclassified phylum) | Chloroplastida | 0.672 | 0.005 |
| unclassified Mediophyceae | Ochrophyta | Stramenopiles | 0.669 | 0.005 |
| unclassified Sphaeropleales | Chlorophyta (unclassified phylum) | Chloroplastida | 0.659 | 0.005 |
| unclassified Cyclopoida | Arthropoda | Metazoa (Animalia) | 0.652 | 0.007 |
| Monodus | Ochrophyta | Stramenopiles | 0.648 | 0.005 |
| Symbiodinium | Dinoflagellata | Alveolata | 0.643 | 0.005 |
| Cyclotella | Ochrophyta | Stramenopiles | 0.642 | 0.005 |
| Thalassiosira | Ochrophyta | Stramenopiles | 0.638 | 0.029 |
| uncultured Rhizophydiales | Chytridiomycota | Fungi | 0.624 | 0.005 |
| unclassified Ochrophyta | Ochrophyta | Stramenopiles | 0.622 | 0.015 |
| Desmodesmus | Chlorophyta (unclassified phylum) | Chloroplastida | 0.619 | 0.005 |
| Scenedesmus | Chlorophyta (unclassified phylum) | Chloroplastida | 0.615 | 0.005 |
| unclassified Pezizomycotina II | Ascomycota | Fungi | 0.614 | 0.005 |
| Marvania | Chlorophyta (unclassified phylum) | Chloroplastida | 0.611 | 0.005 |

| | | | | |
|---|---|---|---|---|
| Rhogostoma | Cercozoa | Rhizaria | 0.610 | 0.017 |
| unclassified Eustigmatales II | Ochrophyta | Stramenopiles | 0.609 | 0.005 |
| unclassified Eustigmatales | Ochrophyta | Stramenopiles | 0.603 | 0.017 |
| unclassified Chlorophyceae | Chlorophyta (unclassified phylum) | Chloroplastida | 0.603 | 0.005 |
| unclassified Chlorellales | Chlorophyta (unclassified phylum) | Chloroplastida | 0.599 | 0.010 |
| Volvox | Chlorophyta (unclassified phylum) | Chloroplastida | 0.597 | 0.005 |
| unclassified Chlamydomonadales | Chlorophyta (unclassified phylum) | Chloroplastida | 0.589 | 0.023 |
| Micractinium | Chlorophyta (unclassified phylum) | Chloroplastida | 0.586 | 0.005 |
| unclassified Peronosporomycetes II | Peronosporomycetes | Stramenopiles | 0.582 | 0.010 |
| Golenkinia | Chlorophyta (unclassified phylum) | Chloroplastida | 0.575 | 0.007 |
| Monoraphidium | Chlorophyta (unclassified phylum) | Chloroplastida | 0.575 | 0.021 |
| unclassified Chlorophycea | Chlorophyta (unclassified phylum) | Chloroplastida | 0.574 | 0.013 |
| unclassified Arthropoda | Arthropoda | Metazoa (Animalia) | 0.570 | 0.005 |
| Tetracystis | Chlorophyta (unclassified phylum) | Chloroplastida | 0.570 | 0.007 |
| Stephanodiscus | Ochrophyta | Stramenopiles | 0.569 | 0.013 |
| Synedra | Ochrophyta | Stramenopiles | 0.568 | 0.007 |
| Polytoma | Chlorophyta (unclassified phylum) | Chloroplastida | 0.567 | 0.017 |
| Aulacoseira | Ochrophyta | Stramenopiles | 0.559 | 0.007 |
| unclassified Rhabditida | Nematoda | Metazoa (Animalia) | 0.557 | 0.013 |
| unclassified Chromadorida | Nematoda | Metazoa (Animalia) | 0.556 | 0.005 |
| Ophryocystis | Apicomplexa | Alveolata | 0.556 | 0.005 |
| unclassified Limnomedusae | Cnidaria | Metazoa (Animalia) | 0.546 | 0.029 |
| unclassified Monhysterida | Nematoda | Metazoa (Animalia) | 0.545 | 0.019 |
| Mychonastes | Chlorophyta (unclassified phylum) | Chloroplastida | 0.544 | 0.048 |
| Nannochloropsis | Ochrophyta | Stramenopiles | 0.542 | 0.015 |
| Chlorococcum | Chlorophyta (unclassified phylum) | Chloroplastida | 0.541 | 0.010 |
| unclassified Chytridiales | Chytridiomycota | Fungi | 0.539 | 0.015 |
| Saprolegnia | Peronosporomycetes | Stramenopiles | 0.539 | 0.023 |
| Blastocystis | Incertae Sedis | Stramenopiles | 0.538 | 0.007 |
| Synura | Ochrophyta | Stramenopiles | 0.537 | 0.010 |
| Stichococcus | Chlorophyta (unclassified phylum) | Chloroplastida | 0.536 | 0.017 |

| | | | | |
|---|---|---|---|---|
| unclassified Hypocreales II | Ascomycota | Fungi | 0.530 | 0.029 |
| unclassified Calanoida | Arthropoda | Metazoa (Animalia) | 0.530 | 0.040 |
| Chlamydomonas | Chlorophyta (unclassified phylum) | Chloroplastida | 0.522 | 0.021 |
| unclassified Chromadorea | Nematoda | Metazoa (Animalia) | 0.515 | 0.019 |
| Fragilaria | Ochrophyta | Stramenopiles | 0.514 | 0.015 |
| Debaryomyces | Ascomycota | Fungi | 0.509 | 0.029 |
| unclassified Nematoda | Nematoda | Metazoa (Animalia) | 0.506 | 0.015 |
| Eustigmatos | Ochrophyta | Stramenopiles | 0.505 | 0.010 |
| Gomphonema | Ochrophyta | Stramenopiles | 0.501 | 0.027 |
| unclassified Chytridiaceae II | Chytridiomycota | Fungi | 0.477 | 0.019 |
| Carteria | Chlorophyta (unclassified phylum) | Chloroplastida | 0.463 | 0.025 |
| Cryptomonas | Cryptomonadales | Cryptophyceae | 0.453 | 0.015 |
| unclassified Mortierellales | Incertae Sedis | Fungi | 0.449 | 0.023 |

**Table S7.** *Pfiesteria* read counts and percentages from different substrates and locations.

| substrate | reads | percent | location | reads | percent |
|---|---|---|---|---|---|
| **PE** | 117566 | 47.9% | **Station 1** | 54 | 0.0% |
| **PS** | 98750 | 40.2% | **Station 2** | 462 | 0.2% |
| **wood** | 25825 | 10.5% | **Station 3** | 83 | 0.0% |
| **water > 0.2 µm** | 681 | 0.3% | **Station 4** | 41794 | 17.0% |
| **water > 3.0 µm** | 2629 | 1.1% | **Station 5** | 203051 | 82.7% |
| | | | **Station 6** | 6 | 0.0% |
| | | | **Station 7** | 1 | 0.0% |
| sum | 245451 | 100.0% | sum | 245451 | 100.0% |

**Table S8.** *Pfiesteria* read counts from the substrate replicates at station 4 and 5.

| substrate | station 4 replicate a | station 4 replicate b | station 4 replicate c | station 5 replicate a | station 5 replicate b | station 5 replicate c |
|---|---|---|---|---|---|---|
| **PE** | 2149 | 1990 | 4606 | 39234 | 22490 | 46730 |
| **PS** | 12684 | 10921 | 6041 | 37222 | 30381 | 1337 |
| **wood** | 1209 | 1460 | 99 | 13466 | 5925 | 3629 |
| **water > 0.2 µm** | 4 | 515 | NA | 36 | 96 | NA |
| **water > 3.0 µm** | 66 | 50 | NA | 2338 | 167 | NA |

**Table S9.** Top 50 hits from NCBI's Web BLAST service (BLASTN 2.6.1, default settings, (Morgulis et al., 2008; Zhang et al., 2000)) of the most abundant sequence that was assigned to the genus *Pfiesteria*.

| Description | Max score | Total score | Query cover | E value | Identity | Accession number |
|---|---|---|---|---|---|---|
| Pfiesteriaceae sp. *masanensis* isolate VIMS 1050 small subunit ribosomal RNA gene, partial sequence; internal transcribed spacer 1, 5.8S ribosomal RNA gene, and internal transcribed spacer 2, complete sequence; and large subunit ribosomal RNA gene, partial sequence | 688 | 688 | 100% | 0.0 | 99% | EU048553.1 |
| Pfiesteriaceae sp. *masanensis* isolate VIMS 1041 small subunit ribosomal RNA gene, partial sequence; internal transcribed spacer 1, 5.8S ribosomal RNA gene, and internal transcribed spacer 2, complete sequence; and large subunit ribosomal RNA gene, partial sequence | 688 | 688 | 100% | 0.0 | 99% | EU048552.1 |
| Dinophyceae sp. Lucy-3 18S small subunit ribosomal RNA gene, complete sequence | 688 | 688 | 100% | 0.0 | 99% | AY251289.1 |
| *Pfiesteria*-like dinoflagellate 18S rRNA gene, ITS1, 5.8S rRNA gene, ITS2 and 28S rRNA gene (partial), strain Jeju Lucy-200504 | 688 | 688 | 100% | 0.0 | 99% | AM050345.1 |
| *Pfiesteria*-like dinoflagellate 18S rRNA gene, ITS1, 5.8S rRNA gene, ITS2 and 28S rRNA gene (partial), strain Masan Lucy-200505 | 688 | 688 | 100% | 0.0 | 99% | AM050344.1 |
| *Pfiesteria*-like sp. clone POC-8 small subunit ribosomal RNA gene, complete sequence | 688 | 688 | 100% | 0.0 | 99% | AY121856.1 |
| *Pfiesteria*-like dinoflagellate 18S small subunit ribosomal RNA gene, partial sequence | 688 | 688 | 100% | 0.0 | 99% | AY033487.1 |
| *Pfiesteria*-like sp. HR1NovC5 small subunit ribosomal RNA gene, partial sequence; internal transcribed spacer 1, 5.8S ribosomal RNA gene, and internal transcribed spacer 2, complete sequence; and large subunit ribosomal RNA gene, partial sequence | 688 | 688 | 100% | 0.0 | 99% | AY590482.1 |
| *Pfiesteria*-like sp. CCMP1835 small subunit ribosomal RNA gene, partial sequence; internal transcribed spacer 1, 5.8S ribosomal RNA gene, and internal transcribed spacer 2, complete sequence; and large subunit ribosomal RNA gene, partial sequence | 688 | 688 | 100% | 0.0 | 99% | AY590477.1 |
| Uncultured eukaryote clone WS073.008 18S ribosomal RNA gene, partial | 682 | 682 | 100% | 0.0 | 99% | KP404756.1 |

| | | | | | | |
|---|---|---|---|---|---|---|
| sequence | | | | | | |
| *Stoeckeria* sp. SSSC09 genomic DNA containing 18S rRNA gene, ITS1, 5.8S rRNA gene, ITS2 and 28S rRNA gene, strain SSSC09 | 682 | 682 | 100% | 0.0 | 99% | HG005132.1 |
| Uncultured eukaryote clone KRL09E10 18S ribosomal RNA gene, partial sequence | 682 | 682 | 100% | 0.0 | 99% | KC315836.1 |
| *Stoeckeria* sp. SSMS0806 18S rRNA gene (partial), ITS1, 5.8S rRNA gene, ITS2 and 28S rRNA gene (partial), strain SSMS0806 | 682 | 682 | 100% | 0.0 | 99% | FN557541.1 |
| Uncultured dinoflagellate clone ssu14 18S ribosomal RNA gene, partial sequence | 682 | 682 | 100% | 0.0 | 99% | AY628342.1 |
| Dinophyceae sp. Shepard's Crook 18S small subunit ribosomal RNA gene, complete sequence | 682 | 682 | 100% | 0.0 | 99% | AY251291.1 |
| Dinophyceae sp. Shepherd's Crook small subunit ribosomal RNA gene, partial sequence; internal transcribed spacer 1, 5.8S ribosomal RNA gene, and internal transcribed spacer 2, complete sequence; and large subunit ribosomal RNA gene, partial sequence | 682 | 682 | 100% | 0.0 | 99% | AY590479.1 |
| *Pfiesteria*-like sp. HR1SSeptA5 small subunit ribosomal RNA gene, partial sequence; internal transcribed spacer 1, 5.8S ribosomal RNA gene, and internal transcribed spacer 2, complete sequence; and large subunit ribosomal RNA gene, partial sequence | 682 | 682 | 100% | 0.0 | 99% | AY590483.1 |
| *Pfiesteria*-like sp. NC Lucy-V27 small subunit ribosomal RNA gene, partial sequence; internal transcribed spacer 1, 5.8S ribosomal RNA gene, and internal transcribed spacer 2, complete sequence; and large subunit ribosomal RNA gene, partial sequence | 680 | 680 | 100% | 0.0 | 99% | AY590485.1 |
| Uncultured eukaryote clone 282A09 small subunit ribosomal RNA gene, partial sequence | 676 | 676 | 100% | 0.0 | 99% | KJ925512.1 |
| Uncultured marine eukaryote clone I-9-MC884-OTU-47 18S ribosomal RNA gene, partial sequence | 676 | 676 | 100% | 0.0 | 99% | KC771146.1 |
| Uncultured eukaryote clone AI5F14RJ2E07 18S ribosomal RNA gene, partial sequence | 676 | 676 | 100% | 0.0 | 99% | GU824700.1 |
| Uncultured eukaryote clone AI3F14RJ1C03 18S ribosomal RNA gene, partial sequence | 676 | 676 | 100% | 0.0 | 99% | GU824602.1 |
| Uncultured eukaryote clone AI5F14RJ1G07 18S ribosomal RNA gene, partial sequence | 676 | 676 | 100% | 0.0 | 99% | GU824184.1 |
| ***Pfiesteria piscicida*** clone Ppi+Rhod_cDNA221 18S ribosomal RNA, partial sequence | 676 | 676 | 100% | 0.0 | 99% | FJ600090.1 |

| | | | | | | |
|---|---|---|---|---|---|---|
| Uncultured alveolate partial 18S rRNA gene, clone 4-H5 | 676 | 676 | 100% | 0.0 | 99% | FN690233.1 |
| ***Pfiesteria piscicida*** strain PPSB27 18S ribosomal RNA gene, internal transcribed spacer 1, 5.8S ribosomal RNA gene, and internal transcribed spacer 2, complete sequence; and 28S ribosomal RNA gene, partial sequence | 676 | 676 | 100% | 0.0 | 99% | DQ991382.1 |
| ***Pfiesteria piscicida*** 18S rRNA gene, 5.8S rRNA gene, 28S rRNA gene (partial), ITS1 and ITS2, strain PPMS0507 | 676 | 676 | 100% | 0.0 | 99% | AM231033.1 |
| ***Pfiesteria piscicida*** 18S rRNA gene, 5.8S rRNA gene, 28S rRNA gene (partial), ITS1 and ITS2, strain PPBS0507 | 676 | 676 | 100% | 0.0 | 99% | AM231028.1 |
| *Paulsenella vonstoschii* 18S rRNA gene | 676 | 676 | 100% | 0.0 | 99% | AJ968729.1 |
| Uncultured dinoflagellate clone ssu34 18S ribosomal RNA gene, partial sequence | 676 | 676 | 100% | 0.0 | 99% | AY628348.1 |
| ***Pfiesteria piscicida*** small subunit ribosomal RNA gene, internal transcribed spacer 1, 5.8S ribosomal RNA gene and internal transcribed spacer 2, complete sequence; and large subunit ribosomal RNA gene, partial sequence | 676 | 676 | 100% | 0.0 | 99% | AY112746.1 |
| ***Pfiesteria piscicida*** isolate NCSU B-125-4(12/9) 18S small subunit ribosomal RNA gene, internal transcribed spacer 1, 5.8S ribosomal RNA gene and internal transcribed spacer 2, complete sequence; and 23S large subunit ribosomal RNA gene, partial sequence | 676 | 676 | 100% | 0.0 | 99% | AF330600.1 |
| ***Pfiesteria piscicida*** 18S small subunit ribosomal RNA gene, partial sequence | 676 | 676 | 100% | 0.0 | 99% | AY033488.1 |
| ***Pfiesteria piscicida*** small subunit ribosomal RNA gene, internal transcribed spacer 1, 5.8S ribosomal RNA gene and internal transcribed spacer 2, complete sequence; and large subunit ribosomal RNA gene, partial sequence | 676 | 676 | 100% | 0.0 | 99% | AY245693.1 |
| ***Pfiesteria piscicida*** small subunit ribosomal RNA gene, partial sequence | 676 | 676 | 100% | 0.0 | 99% | AF149793.1 |
| ***Pfiesteria piscicida*** small subunit ribosomal RNA gene, complete sequence | 676 | 676 | 100% | 0.0 | 99% | AF077055.1 |
| Uncultured eukaryote clone 268D02 small subunit ribosomal RNA gene, partial sequence | 675 | 675 | 100% | 0.0 | 99% | KJ925188.1 |
| Uncultured eukaryote clone KRL03E38 18S ribosomal RNA gene, partial sequence | 675 | 675 | 100% | 0.0 | 99% | KC315816.1 |
| Uncultured dinoflagellate clone SSU1_M0 18S ribosomal RNA gene, partial sequence | 675 | 675 | 100% | 0.0 | 99% | AY628349.1 |

**Fig. S3.** Co-occurrence networks for PE (A, B), PS (C, D) and wood (E, F) for both incubation experiments I (Baltic Sea to River Warnow, stations 1 to 5) and II (WWTP, stations 6 and 7). Networks were calculated in Cytoscape 3.5.1 (Shannon et al., 2003) with the CoNet app version 1.1.1. beta (Faust and Raes, 2016). Nodes are labelled with taxon name and relative node sizes correspond to number of neighbor nodes. Colors indicate different kingdoms.

Archaea | Bacteria | Alveolata | Chloroplastida
Cryptophyceae | Fungi | unclassified kingdom in Holozoa
Metazoa (Animalia) | Rhizaria | Rhodophyceae | Stramenopiles

**Fig. S1 A) PE co-occurrence network (stations 1 to 5)**

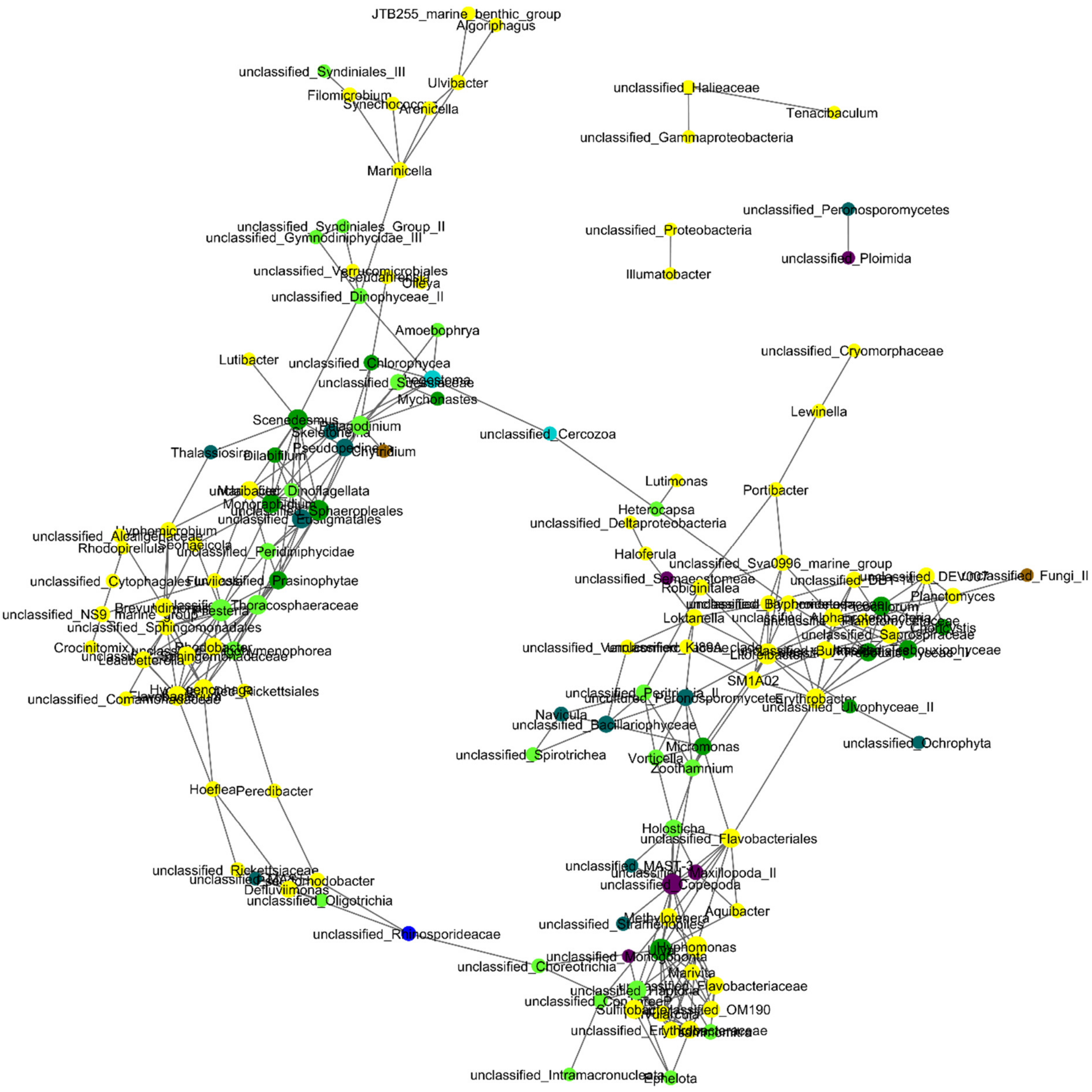

**Fig. S1 B) PE co-occurrence network (stations 6 and 7)**

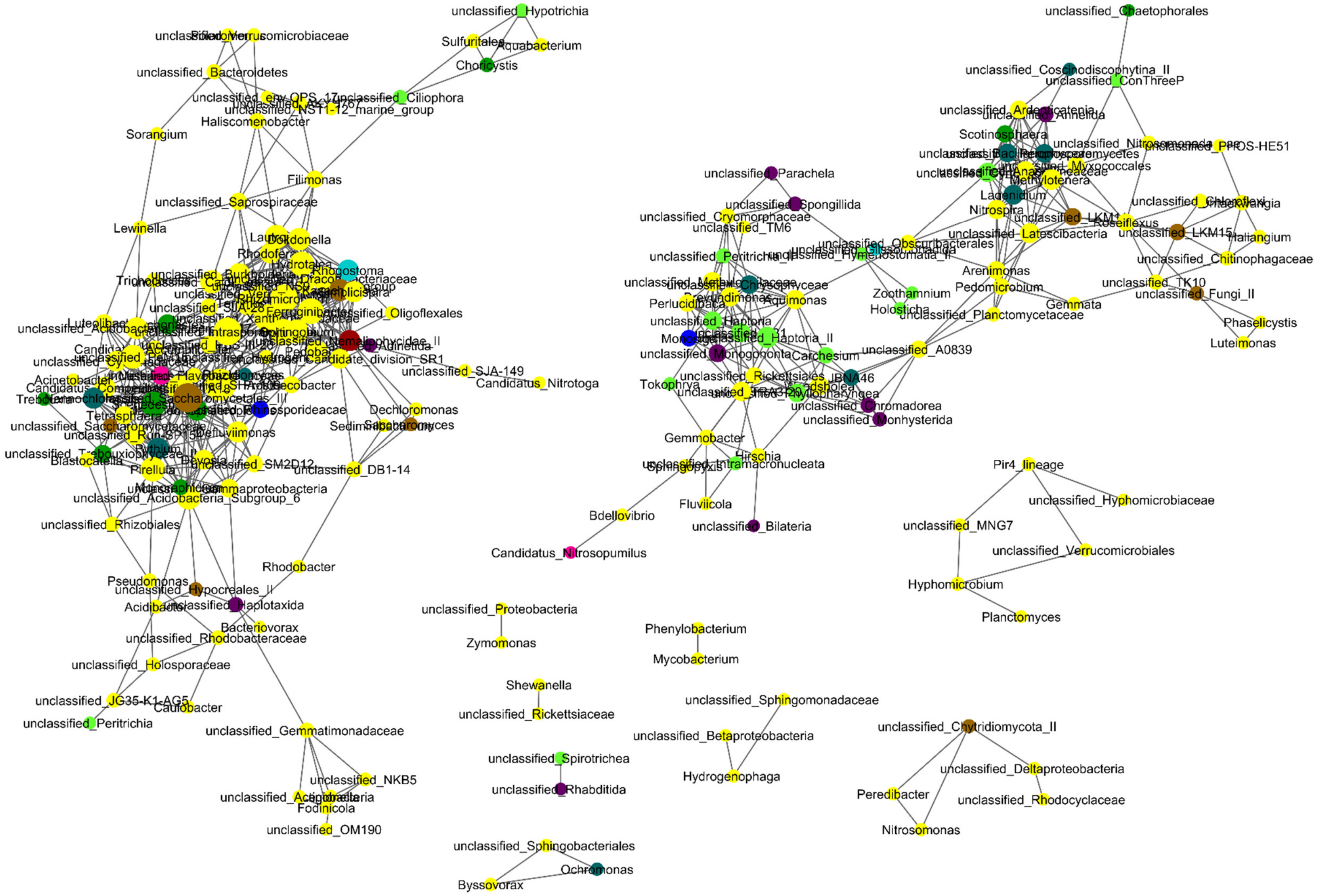

**Fig. S1 C) PS co-occurrence network (stations 1 to 5)**

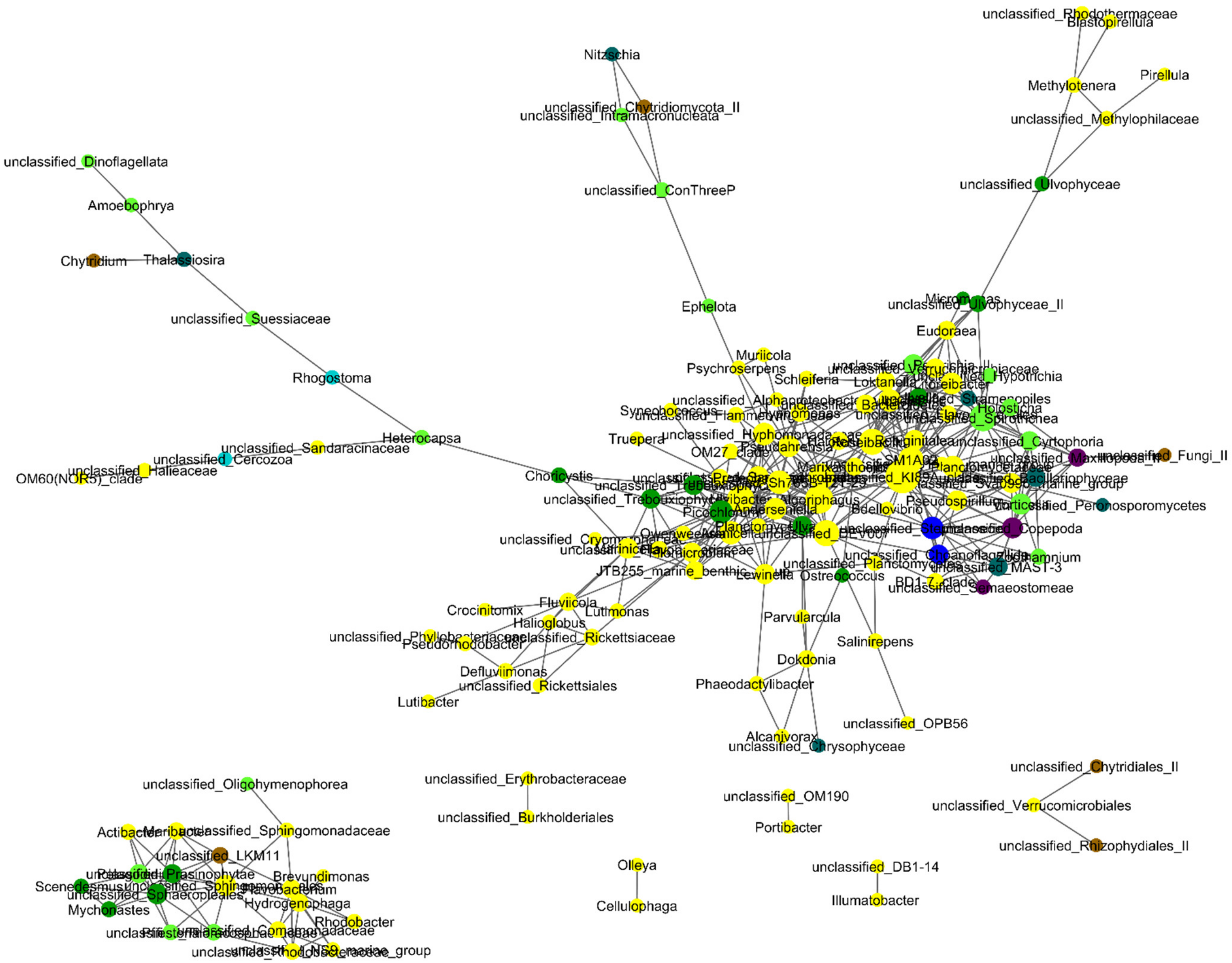

**Fig. S1 D) PS co-occurrence network (stations 6 and 7)**

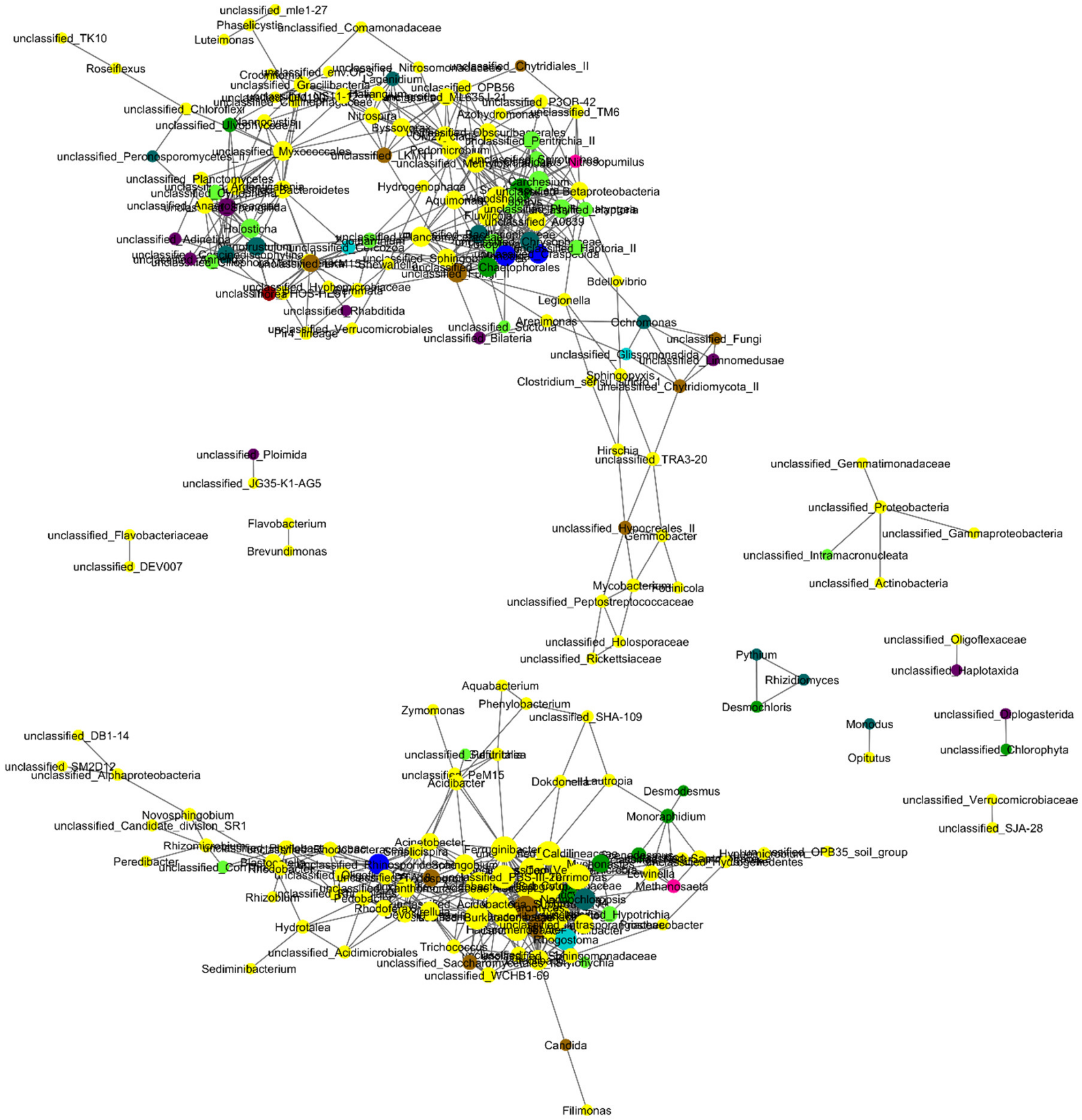

**Fig. S1 E) wood co-occurrence network (stations 1 to 5)**

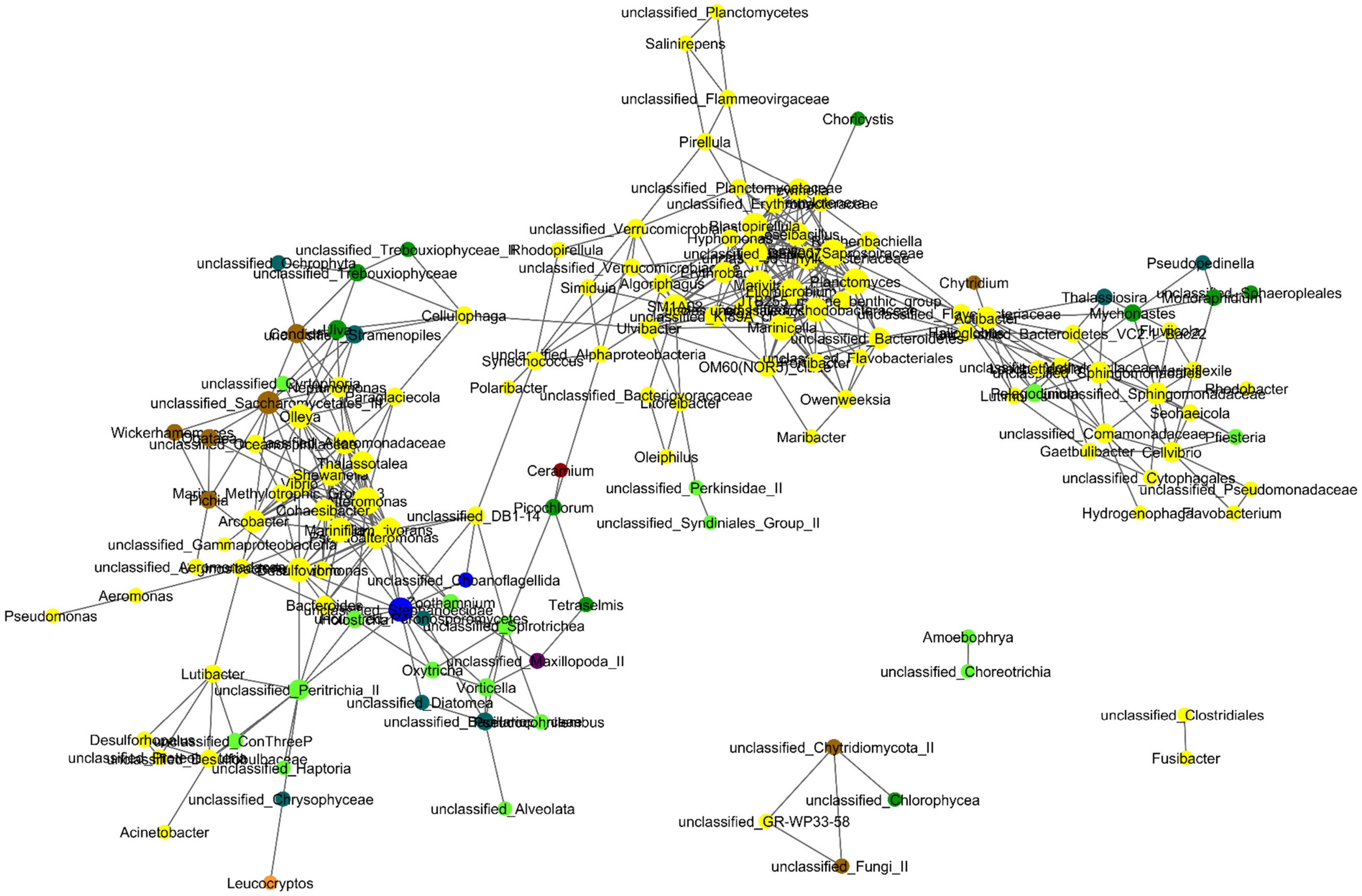

## Fig. S1 F) wood co-occurrence network (stations 6 and 7)

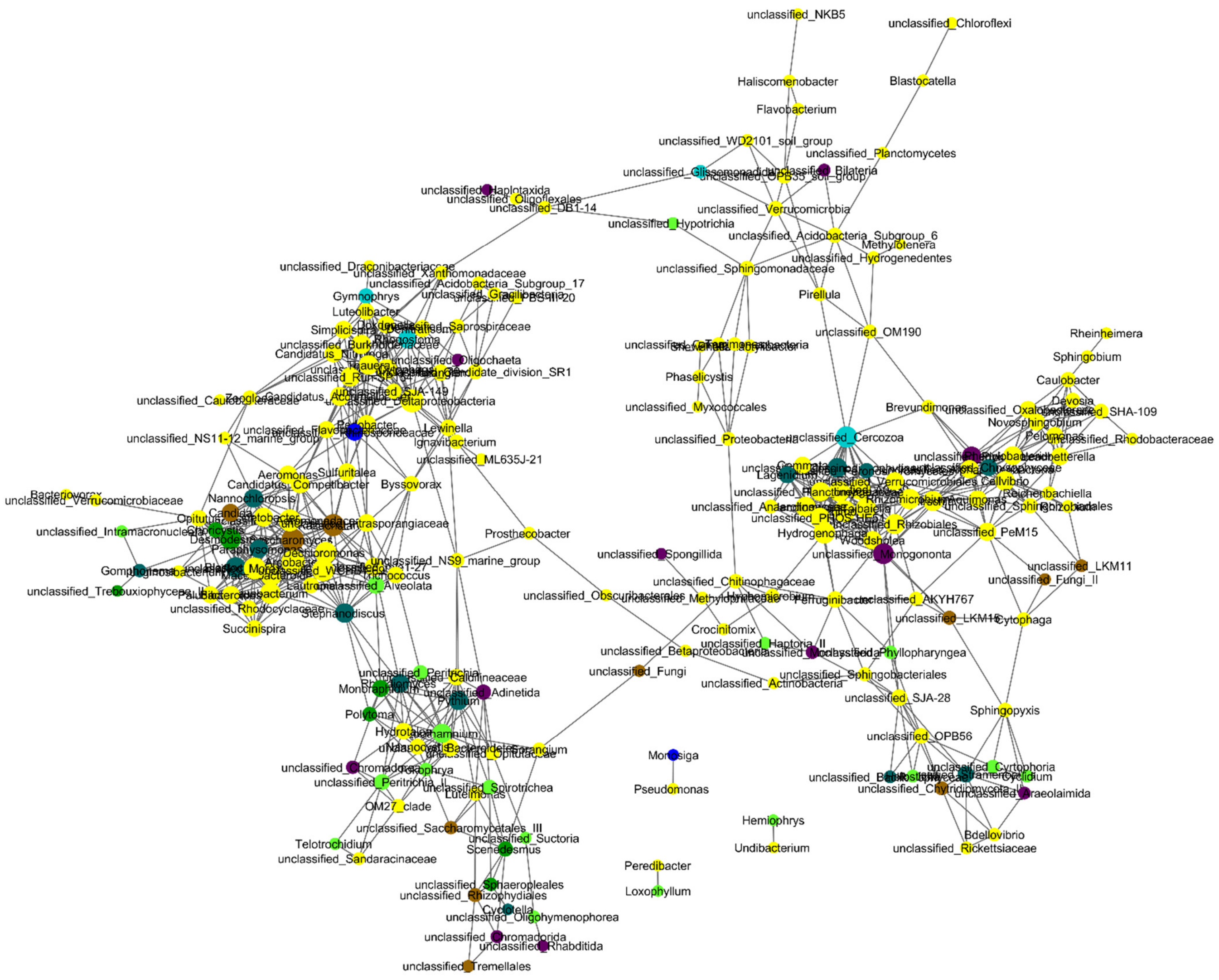

*References*


Benjamini, Y., and Hochberg, Y. (1995). Controlling the false discovery rate: a practical and powerful approach to multiple testing. *J. R. Stat. Soc. Ser. B* 57, 289–300. Available at: http://www.jstor.org/stable/2346101.

Faust, K., and Raes, J. (2016). CoNet app: inference of biological association networks using Cytoscape. *F1000Research* 5, 1519. doi:10.12688/f1000research.9050.1.

Kettner, M. T., Rojas-Jimenez, K., Oberbeckmann, S., Labrenz, M., and Grossart, H.-P. (2017). Microplastics alter composition of fungal communities in aquatic ecosystems. *Environ. Microbiol.* 19, 4447–4459. doi:10.1111/1462-2920.13891.

Klindworth, A., Pruesse, E., Schweer, T., Peplies, J., Quast, C., Horn, M., et al. (2013). Evaluation of general 16S ribosomal RNA gene PCR primers for classical and next-generation sequencing-based diversity studies. *Nucleic Acids Res.* 41, 1–11. doi:10.1093/nar/gks808.

Morgulis, A., Coulouris, G., Raytselis, Y., Madden, T. L., Agarwala, R., and Schäffer, A. A. (2008). Database indexing for production MegaBLAST searches. *Bioinformatics* 24, 1757–1764. doi:10.1093/bioinformatics/btn322.

Shannon, P., Markiel, A., Ozier, O., Baliga, N. S., Wang, J. T., Ramage, D., et al. (2003). Cytoscape: a software environment for integrated models of biomolecular interaction networks. *Genome Res.* 13, 2498–504. doi:10.1101/gr.1239303.

Stoeck, T., Bass, D., Nebel, M., Christen, R., Jones, M. D. M., Breiner, H.-W., et al. (2010). Multiple marker parallel tag environmental DNA sequencing reveals a highly complex eukaryotic community in marine anoxic water. *Mol. Ecol.* 19, 21–31. doi:10.1111/j.1365-294X.2009.04480.x.

Yilmaz, P., Parfrey, L. W., Yarza, P., Gerken, J., Pruesse, E., Quast, C., et al. (2014). The SILVA and "all-species Living Tree Project (LTP)" taxonomic frameworks. *Nucleic Acids Res.* 42, 1–6. doi:10.1093/nar/gkt1209.

Zhang, Z., Schwartz, S., Wagner, L., and Miller, W. (2000). A Greedy Algorithm for Aligning DNA Sequences. *J. Comput. Biol.* 7, 203–214. doi:10.1089/10665270050081478.
